# Construct Validity of Frontier Large Language Models on 4 Spanish Health Professions Specialty-Access Examinations (MIR, EIR, PIR, and FIR): Cross-Sectional Benchmarking and Psychometric Study

**DOI:** 10.2196/106199

**Published:** 2026-10-29

**Authors:** Clemente García-Hidalgo, Manuel Carpio-Salmerón, Pau Benito, Carlos Carazo-Casas, Beatriz Carratalá, Jaime Campos Pavón

**Affiliations:** 1 Radiology Department, Hospital General Universitario Morales Meseguer, Av Marqués de los Vélez S/N, Murciaº, Murcia, 30008, Spain, 34 666817007; 2 Endocrinology Department, Santa Lucía University General Hospital, Cartagena, Murcia, Spain; 3 Department of Preventive Medicine and Epidemiology, Clinical Institute of Medicine and Dermatology (ICMiD), Hospital Clínic de Barcelona, Barcelona, Catalonia, Spain; 4 Barcelona Institute for Global Health (ISGlobal), Universitat de Barcelona, Hospital Clínic de Barcelona, Barcelona, Catalonia, Spain; 5 Department of Otolaryngology, Hospital Universitario Ramón y Cajal, Madrid, Madrid, Spain; 6 Innovation and Digital Projects Academic Department, Healthcademia, Madrid, Madrid, Spain; 7 Academia AMIR, Madrid, Madrid, Spain; 8 Department of Ophthalmology, Hospital Universitario, Madrid, Spain

**Keywords:** large language models, AI, medical education, educational measurement, clinical competence, psychometrics, construct validity, specialty-access examination, multiple-choice, Spain

## Abstract

**Background:**

Large language models (LLMs) achieve high scores on health-professions examinations. Whether these scores support the same interpretations as human scores remains uncertain. Spain uses parallel multiple-choice examinations for entry to specialty training in medicine (Médico Interno Residente [MIR]), nursing (Enfermero Interno Residente [EIR]), psychology (Psicólogo Interno Residente [PIR]), and pharmacy (Farmacéutico Interno Residente [FIR]).

**Objective:**

Using the January 2026 MIR, EIR, PIR, and FIR examinations, this study aimed to quantify the correspondence between human and LLM item facility and to describe model accuracy, distractor selection, and performance associated with measured surface features.

**Methods:**

We evaluated 839 valid items using 12 configurations: GPT-5.2 at 3 reasoning efforts and Claude Opus, Sonnet, and Haiku 4.5 (Anthropic), each under neutral and expert prompts, with one run per configuration. The primary measure was the Spearman correlation (ρ) between human and machine facility on 828 complete paired items. We examined sensitivity to panel composition, scoring, pooling, and assumed reliability; compared distractor choices with a post hoc plausibility-weighted baseline; and evaluated a surface-feature classifier. Image analyses distinguished supplied from withheld images. An exploratory LLM-judge audit assessed generated explanations. Bootstrap CIs resample items or, for distractor concordance, error events; they exclude run-to-run uncertainty.

**Results:**

The highest pooled accuracy was 94.8% (795/839); all 12 configurations answered 668 of 828 paired items correctly (80.7%). Scores clustered near the ceiling among the strongest configurations. Human and machine facility correlated at 0.40 (95% CI 0.34-0.45), with estimates of 0.31 to 0.40 across panel definitions and 0.39 (95% CI 0.33-0.45) after exam-stratified pooling. The observed correlation was lower than predicted by a specified one-axis simulation with a uniform model advantage (0.63; predictive interval 0.59 to 0.66; *P*<.001). Among 672 model errors, dominant human-distractor selection differed from the plausibility-weighted baseline by –1.25 percentage points (95% CI –4.53 to 2.19), compatible with no difference. Surface-feature accuracy was 34.4% (289/839), similar to selecting the longest option (34.6%, 290/839). The adjusted association with model correctness was uncertain (odds ratio [OR] 0.87, 95% CI 0.72-1.04). Image-associated accuracy was lower both when images were supplied (OR 0.33, 95% CI 0.21-0.53) and when withheld (OR 0.11, 95% CI 0.08-0.16).

**Conclusions:**

In this single 2026 cohort, high LLM scores coexisted with limited separation among the strongest configurations and moderate correspondence with human item difficulty. These findings caution against interpreting model scores as equivalent evidence of human competence. They do not establish clinical performance or invalidate the examinations for human candidates.

## Introduction

### Background

Large language models (LLMs) are increasingly used in health-professions education. Reviews identify potential benefits, including personalized tutoring, virtual patients, formative feedback, and support for self-regulated learning. They also describe hallucinations, bias, factual errors, reduced critical thinking, over-reliance, and threats to academic integrity [1-3]. In a review of 33 studies, Pham et al [3] found that generative AI (GenAI) mainly supported individual activities, with little use in collaborative discussion. These tools may provide educational scaffolding [4-7], but high examination scores alone do not establish whether they support or replace the learning processes an examination is intended to assess.

LLM performance on medical multiple-choice questions (MCQs) has improved rapidly. ChatGPT (GPT-3.5; OpenAI) reached the United States Medical Licensing Examination (USMLE) passing threshold [8]; GPT-4 exceeded it by over 20 points and showed better calibration [9]. A meta-analysis of 45 studies across 17 countries reported pooled accuracy of 81% for GPT-4 and 58% for GPT-3.5, while concluding that GPT-4 was not yet suitable for unsupervised use [10]. Studies of Spain’s Médico Interno Residente (MIR) examination have reported a similar progression, from approximately 51% for GPT-3.5 [11] to passing performance for GPT-4 [12,13] and subsequent evaluations of GPT-4o and o1 [14,15]. Other benchmarks assess LLM clinical reasoning using USMLE-style scenarios [16].

A high score and a valid interpretation of that score are separate claims. Construct-validity theory asks whether evidence supports the intended interpretation and use of test scores. Messick [17] identified 2 main threats: construct underrepresentation, when a test captures too little of the intended competence, and construct-irrelevant variance, when other factors affect scores. Kane’s [18] approach examines a chain of inferences from scoring and generalization to extrapolation and decisions, with particular attention to the weakest links [19]. The Standards for Educational and Psychological Testing describes 5 sources of validity evidence and identifies fairness as a basic requirement [20,21]. Comparing score interpretations across populations also requires attention to measurement invariance and its item-level counterpart, differential item functioning (DIF) [22].

These examinations were designed for human candidates. Evidence supporting their use for human selection does not automatically support the same interpretation for an LLM, whose prior exposure to examination material and response processes may differ. High model accuracy therefore does not, by itself, establish professional competence.

### Prior Work

Recent studies apply construct-validity theory to AI benchmarks. Raji et al [23] caution against treating benchmark scores as measures of general capability. In a review of 445 LLM benchmarks, Bean et al [24] found that approximately 47.8% assessed contested constructs and 53.4% provided any construct-validity evidence. Eriksson et al [25] identified weak construct validity among 9 recurring concerns, including contamination and saturation. Similar concerns have been raised for medical LLM benchmarks, where MCQ accuracy alone may not support the intended interpretation of performance [26].

Shortcut learning and data contamination may affect the meaning of benchmark scores. Shortcut learning uses superficial cues that predict answers within a dataset but may fail in other settings [27]. Examples include the Clever Hans problem in computer vision [28], pneumonia classifiers using hospital-specific image features [29], and similar concerns in COVID-19 imaging. A scoping review of 173 studies found that most did not test for shortcuts [30,31]. LLMs can answer MCQs above chance after the question stem is removed [32], and contamination studies report leakage as high as approximately 91.8% and lower scores after items are rewritten [33,34]. Correct answers may also draw on abilities different from those intended by an examination. About a third of medical question-answering items require multistep reasoning [35]; studies report difficulty recognizing unanswerable questions [36], failure on Einstellung vignettes that physicians solve [37], and explanations that do not faithfully reflect the process producing an answer [38].

Spain provides a common examination format across 4 health professions. The MIR examination ranks candidates for specialty training using approximately 200 anonymized MCQs with 4 options and 3 points for a correct answer and a penalty of 1 point for an incorrect answer. scoring, combined with an academic-record score [39]. Nursing (Enfermero Interno Residente [EIR]), psychology (Psicólogo Interno Residente [PIR]), and pharmacy (Farmacéutico Interno Residente [FIR]) use parallel examinations. This allows comparison across 4 knowledge domains within 1 selection system, although these examinations should not be treated as independent tests of different assessment designs. The 2026 Formación Sanitaria Especializada (FSE) call offered 12,366 places, including 9276 in medicine, with 34,553 candidates admitted [39,40]. The Ministry projects a specialist-workforce shortage approaching 9000 around 2027, particularly affecting an aging family and community medicine workforce, despite recurrent unfilled training places in that specialty [41]. High model examination scores do not address these workforce distribution problems. Spain’s reliance on a single MCQ ranking examination also differs from assessment systems used elsewhere [42-47]. We did not evaluate non-Spanish items or test whether more varied assessment formats are more resistant to AI-assisted answering.

The literature leaves 3 questions for this study. First, do frontier LLMs show similar performance across medicine, nursing, psychology, and pharmacy specialty-access examinations? Second, do model scores support the same interpretation as human scores at the item level, considering difficulty, distractor choices, surface cues, and the explanations models provide? Third, do these patterns occur across the 4 professions within a common examination format? The January 2026 examinations and their subsequently released answer keys provide a recent cohort for this comparison. Their timing does not establish that the tested models had no prior exposure to the items or to similar examination content.

### Objectives

Using the January 2026 MIR, EIR, PIR, and FIR examinations, our primary objective was to quantify the correspondence between human item facility and the proportion of 12 LLM configurations answering each item correctly, using Spearman correlation (ρ) on complete paired items. Secondary objectives were to describe accuracy across the 839 valid items, compare model and human distractor choices, and assess whether measured surface features were associated with model correctness. Analyses of generated explanations, calibration, latency, image administration, and stylometry were exploratory. These outcomes inform an interpretation of examination scores within Kane validity framework; they do not constitute a formal test of measurement invariance or clinical competence.

## Methods

### Study Design and Validity Framework

We conducted a cross-sectional, retrospective study of frontier LLM performance on the 4 Spanish national specialty-access examinations in the 2026 FSE call: medicine (MIR), nursing (EIR), psychology (PIR), and pharmacy (FIR). Each examination uses negatively marked, 4-option MCQs. The examination score and academic record determine a national ranking for entry to specialty training [11,39]. We evaluated the interpretation of model scores using Messick construct-validity framework and Kane argument-based approach, as applied by Cook et al [17-20]. For model responses, we examined evidence concerning scoring, generalization, and extrapolation, together with construct-irrelevant variance and construct underrepresentation. Table 1 maps these inferences to the evidence and its limits.

**Table 1.** Evidence for interpreting model examination scores within Kane framework^a^.

Kane inference	Evidence in this study	Scope of inference for models
Scoring	Exact agreement of the parsed option with the official key; incomplete responses examined separately.	Supported for recorded answers against the official key.
Generalization	Four professions within one examination design; related model configurations; one run each; 668/828 items at the panel ceiling.	Evidence limited to these items and configurations. Generalization to new occasions, prompts, and test forms remains uncertain.
Extrapolation	Moderate human-machine item-facility correspondence; generated explanations; no person-level invariance or clinical outcomes.	Clinical competence and equivalence with human score interpretations are untested.
Decision	High accuracy and limited separation among the strongest configurations; exploratory, unvalidated audit labels.	The study does not validate clinical decisions or autonomous educational use.

a These judgments describe the scope of the current evidence; they do not evaluate the examinations’ validity for human candidates.

We organized the exploratory analyses into five areas: (1) agreement between human and machine item difficulty, (2) agreement in errors and distractor choices, (3) whether surface features predict the correct answer and are associated with model accuracy, (4) an LLM-judge audit of errors and explanations, and (5) the writing style of model explanations, reported as supporting analysis in Part S1 in Multimedia Appendix 1. We organized the exploratory analyses into five areas: (1) agreement between human and machine item difficulty, (2) agreement in errors and distractor choices, (3) prediction of the correct answer from surface features and the association of these features with model accuracy, (4) an LLM-judge audit of errors and explanations, and (5) the writing style of model explanations. The fifth area is reported as a supporting analysis in Part S1 in Multimedia Appendix 1. Interpretations concerning recall, reasoning, and potentially harmful answers remained provisional because they depended on the exploratory audit.

We distinguish expert agreement with the official key (n/N, 88% to n/N, 97%) from candidate facility, the proportion of examinees answering correctly. These measures have different denominators, defined in Results section. Comparisons with the average candidate use candidate facility only. Neither expert key agreement nor candidate or model MCQ accuracy is treated as a direct measure of clinical performance. Clinical competence is discussed as an untested extrapolation from examination performance.

### Data Sources and Item Construction

We used official question booklets and answer keys from the Ministerio de Sanidad. Items required an official key and could not be annulled, giving 839 valid items (MIR: n=203, EIR: n=220, PIR: n=210, and FIR: n=206). Image-associated items were flagged (MIR: n=24, EIR: n=20, PIR: n=0, and FIR: n=14). From aggregate candidate option distributions, we calculated human facility, normalized Shannon response entropy, the modal option, and the most frequently selected incorrect option. PIR facility was reconstructed from these distributions; we also analyzed MIR, EIR, and FIR separately as the examinations with published candidate facility. Human facility was available for 829 items, including all 210 PIR items. A total of 10 EIR items lacked usable candidate distributions. Claude Opus 4.5 (Anthropic) expert responses were unparsed on those same 10 EIR items and on one further PIR item. The primary analysis required human facility and all 12 model responses, leaving 828 complete paired items (MIR 203, EIR 210, PIR 209, and FIR 206). The observation-level generalized linear mixed model (GLMM) retained the additional PIR item with 11 parsed responses. Figure 1 shows these analysis samples.

**Figure 1.**
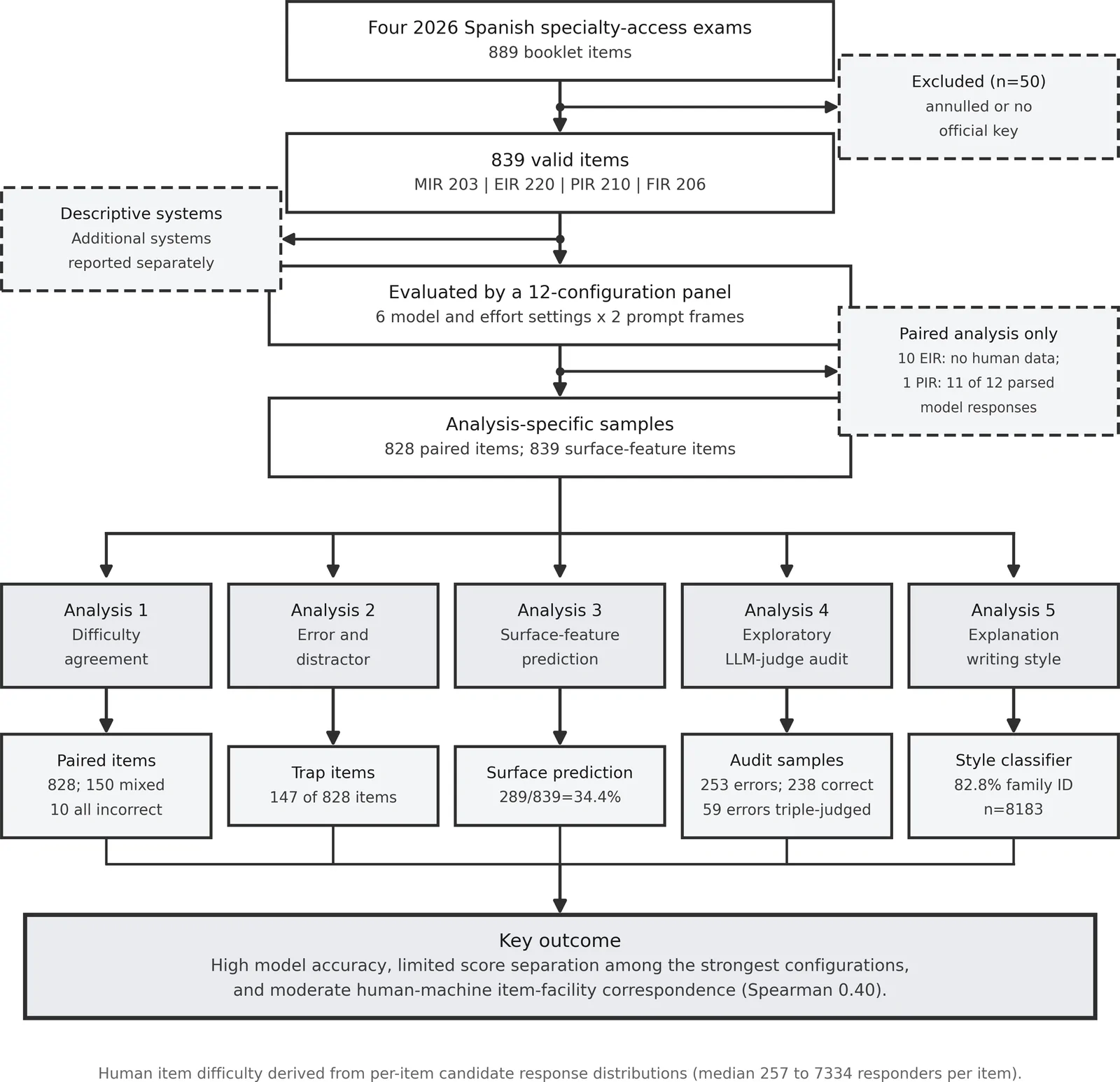
Study design, item selection, and analysis samples for the 4 Spanish specialty-access examinations. EIR: Enfermero Interno Residente; FIR: Farmacéutico Interno Residente; MIR: Médico Interno Residente; PIR: Psicólogo Interno Residente.

### AI Systems, Configurations, and Single-Run Design

The primary panel contained 12 configurations: GPT-5.2 at low, medium, and high reasoning effort and Claude Opus 4.5, Sonnet 4.5, and Haiku 4.5, each tested with neutral and expert prompt frames (6×2=12). GPT-5.2 was accessed through the OpenAI API. Machine facility was the proportion of panel configurations answering an item correctly, with unparsed responses handled as described in the sensitivity analysis. Google Gemini 3 Flash, an MIR-only Claude Opus 4.6 configuration with partial coverage, OpenEvidence, and a funder domain chatbot were evaluated descriptively and excluded from the primary panel and the first 4 validity analyses (see Management of Competing Interests section). Each configuration was run once, without repeated sampling or temperature variation, so run-to-run variance was not estimated. An automated pipeline processed text items and extracted the selected option, explanation, and latency. MIR image items were administered manually to multimodal models with the image. EIR and FIR image items went through the text-only pipeline without the image. Because image availability was confounded with examination, we analyzed these settings separately. An answer was correct only if the parsed option matched the official key. A verification prompt elicited certainty from 1 to 100. Model identifiers, query dates, prompts, decoding parameters, and rules for malformed or abstaining responses are provided in Part S1 in Multimedia Appendix 1.

### Relationship Between Human and Machine Item Difficulty

We applied classical item analysis to the LLM configuration panel. For each item, machine facility was the proportion of configurations answering correctly, and machine discrimination was the point-biserial correlation between item correctness and overall accuracy across configurations. We calculated Kuder-Richardson Formula 20 (KR-20) for each examination. These statistics describe the 12-configuration panel and should not be interpreted as reliability estimates for human candidates. We measured the relationship between human and machine facility using Spearman ρ, overall and by examination, with bootstrap 95% CIs. Squared Pearson correlation (*r*) was reported only as a descriptive shared-variance index. To examine the effect of near-ceiling model accuracy, we used 3 additional analyses: Pearson *r* corrected for assumed measurement reliability; machine facility across deciles of human facility, summarized with an ordinary least-squares slope rather than a fitted DIF model; and a logistic GLMM of response correctness with standardized human facility (per SD) and an image indicator as predictors and item- and model-level random intercepts. The GLMM included 829 items with defined human facility: 828 contributed 12 configurations and 1 contributed 11, yielding 828×12+11=9947 observations.

Machine facility is a coarse proportion based on 12 configurations, with per-item SE up to approximately 0.15. It was also near the ceiling: all configurations answered 668 of 828 items correctly (80.7%). These features can reduce the observed correlation with human facility. We assessed this explanation using a simulation with one shared latent item-difficulty axis. In the one-parameter logistic (1PL) model, human facility determined item difficulty, while the AI panel had a uniform ability advantage of Δ logits and performance was constrained by the empirical ceiling. We calibrated Δ to match observed mean machine facility, not the observed correlation. Across 2000 simulations, we compared the observed Spearman ρ and extreme-cell counts with the model’s predictive distribution. We repeated the simulation using 2PL and 3PL parameterizations and a±1-logit range of Δ. This analysis tests the specified shared-difficulty, uniform-advantage model; it cannot exclude all unidimensional explanations. Full details and sensitivity analyses are mentioned in Part S1 in Multimedia Appendix 1.

### Sensitivity Analyses for Human-Machine Difficulty Agreement

We performed 4 sensitivity analyses using existing item-level data, without new model queries. We recalculated the human-machine correlation and the proportion of items answered correctly by every configuration under 7 panel definitions, listed in Results section. We compared 3 rules for unparsed responses: exclusion from the machine-facility denominator, scoring as incorrect, and complete-case analysis. We estimated correlations within each examination with bootstrap CIs, then combined them using a fixed-effect Fisher-z meta-analysis with Q and *I*^2^. Finally, we varied assumed human-facility reliability from 0.90 to 0.98 and machine-facility reliability from 0.80 to 0.95 when correcting correlations for measurement error.

### Error and Distractor Concordance Between Models and Humans

We defined a trap item as one on which the most frequently selected human option was incorrect. We compared machine facility on trap and nontrap items using bootstrap CIs. Across the 828 paired items, we cross-tabulated trap status against whether an item was difficult for humans but answered correctly by every model configuration. We reported the phi coefficient and human and machine facility in each of the 4 cells to assess whether these definitions identified the same items. Among model errors, we calculated the probability of selecting the human top distractor or the most frequently selected human option, and the mean within-item Pearson *r* between human and model distributions across the 3 incorrect options. We also calculated the Spearman ρ between human response entropy and model certainty. Because incorrect human responses were not evenly distributed across options, we supplemented the uniform-among-wrong baseline of 0.33 with a plausibility-weighted baseline based on the human-choice shares of incorrect options. This post hoc analysis was adopted as the primary comparison for error concordance. We reported observed concordance minus that baseline with a 2000-replicate bootstrap 95% CI resampling individual error events. This interval does not account for dependence among errors from the same item or related configurations. The derivation is mentioned in Part S1 in Multimedia Appendix 1.

### Surface Features and Correct-Answer Prediction

We tested whether predefined surface features could predict the correct option without requiring medical knowledge, an MCQ analog of shortcut learning [27,28,32]. An L2-regularized logistic classifier used 11 features, including option length, position, absolute terms, negation, numerals, all or none-of-the-above patterns, and lexical overlap with the stem. The overlap feature used words from the stem, so the classifier was not entirely independent of stem-derived information. We used leave-one-examination-out cross-validation and reported item-level accuracy overall and by examination, with a bootstrap 95% CI (n=839). All options from each item remained within the same training or test partition. We compared performance with a modal-position baseline and a longest-option rule, and also used within-examination cross-validation. To assess whether these cues were associated with model performance, we compared full-coverage model accuracy between items classified as shortcut-solvable and shortcut-resistant, adjusting for human facility because shortcut-solvable items were also easier for humans. The full feature list is mentioned in Part S1 in Multimedia Appendix 1.

### Exploratory Audit of Errors and Model Explanations

LLM judges applied a human-authored rubric to model explanations, returning labels in a fixed JSON schema. This exploratory audit assessed the generated text, not internal model computation. The labels were unvalidated judgments from the same model family. The rubric and expert prompt were written for a physician examiner despite the 4-profession sample; clinician adjudication would be required before drawing safety conclusions. We performed 2 audits. First, we assigned 1 primary failure mode from 8 categories to each frontier wrong answer with a usable explanation (n=253; Claude Opus, Sonnet, and Haiku 4.5 and GPT-5.2 high, neutral). An answer was flagged as potentially clinically dangerous if following it instead of the official answer could plausibly harm a patient. Second, a stratified sample of correct GPT-5.2 high answers was labeled for faithfulness, dominant expressed reasoning mode, and false statements (240 sampled, 238 labeled, and approximately 60 per examination). Analyses used the 238 labeled answers. We assessed agreement separately using 3-judge Fleiss kappa (κ) in 59 MIR and EIR answers (κ=0.76; 2-of-3 agreement 0.97) and Opus-versus-Sonnet Cohen κ for each label on jointly labeled responses (231 errors and 236 correct-answer rationales). The rubric is mentioned in Part S3 in Multimedia Appendix 1; agreement details and limitations are mentioned in Part S1 in Multimedia Appendix 1. Judge prompts and the rubric are deposited as described in Data Availability section.

### Writing Style of Model Explanations (Supporting Analysis)

We assembled 12,043 AI explanations and 839 human examination stems to describe writing style and trained a random-forest classifier to identify the generating model family from stylistic features. The exploratory analysis is detailed in Part S1 in Multimedia Appendix 1, with one summary panel in the main text. It does not establish whether any official examination item was written by AI.

### Certainty, Latency, Image Availability, and Prompt Frames

We assessed stated certainty (1 to 100) using the area under the receiver operating characteristic curve (AUROC), mean certainty minus accuracy to describe net over- or under-confidence, and expected calibration error (ECE). We recorded median response latency for each configuration. Image associations were estimated in 2 regression models: ordinary logistic regression adjusted for human facility, yielding an odds ratio without item or configuration random effects, and the GLMM described above, yielding a conditional β (image) coefficient with item- and model-level random intercepts. These estimates have different interpretations and are reported separately. Because images were supplied for MIR but withheld for EIR and FIR, we fitted the ordinary logistic model separately for these settings and for the pooled data. Repeating queries with different seeds or temperatures was not feasible in the study environment. We therefore compared the panel-level human-machine correlation and per-model accuracy between neutral and expert prompt frames as a limited measure of prompt sensitivity. This comparison does not estimate run-to-run variation at a fixed prompt.

### Statistical Analysis and Single-Run Uncertainty

We used Python (Python Software Foundation) with statsmodels, scikit-learn, and SciPy. Accuracy is reported as a percentage with its count, and principal estimates are accompanied by bootstrap or binomial 95% CIs. *P* values are descriptive and unadjusted for multiple comparisons. Rank associations use Spearman ρ with item-bootstrap CIs; squared Pearson *r* values are descriptive shared-variance indices. Item-level associations use logistic regression, including McFadden pseudo-*R*², and a GLMM with item- and model-level random intercepts. We interpret nonsignificant contrasts through their CIs rather than as evidence of no effect. KR-20 (0.86 to 0.95) and point-biserial discrimination describe the 12-configuration panel. Each configuration was evaluated once, so KR-20, discrimination, machine facility, the 80.7% ceiling, and ρ=0.40 are conditional on those runs. Most bootstrap CIs resample items; the distractor-concordance interval resamples individual error events and does not account for within-item or configuration dependence. Neither approach includes uncertainty from repeated model responses. They therefore do not capture total uncertainty.

### Management of Competing Interests

The funder (Healthcademia) operates one evaluated domain chatbot. That chatbot is excluded from the 12-configuration headline panel, from all comparative ranking, and from construct-validity pillars 1 to 4; it appears only as a separate, nonranked family in the descriptive stylometric corpus (pillar 5), and its accuracy is reported separately for transparency (Results section): pooled 80.4%; per-examination MIR 86.6%, EIR 85.9%, PIR 74.0%, and FIR 75.4%.

### Ethical Considerations

This work was a secondary analysis of publicly released examination materials and deidentified aggregate candidate statistics. No human participants were recruited or enrolled, no individually identifiable data were accessed, and no model was fine-tuned; institutional review board approval or exemption was therefore not required under institutional and local policy. No participant compensation was provided. The official examination items are the property of the Ministerio de Sanidad and are publicly released after each call, we reference them to that public source rather than redistributing them.

## Results

### Item Flow

Figure 1 summarizes item selection and the separate analysis samples. Of 889 booklet items, 839 had a valid official key and were not annulled. Human facility was available for 829 items; the 10 missing distributions were all from EIR. One further item, from PIR, had only 11 parsed model responses, leaving 828 items with human facility and all 12 model responses for the primary paired analysis. Of these, 668 of 828 (80.7%) were answered correctly by every configuration and 160 of 828 (19.3%) had at least one error. The latter comprised 150 items with both correct and incorrect responses and 10 missed by every configuration. The audit separately assessed 253 incorrect responses and 238 correct-answer rationales; a 59-error MIR and EIR subset underwent triple-judge assessment.

### Model Accuracy and Discrimination Across the 4 Examinations

Across the 4 2026 Spanish specialty-access examinations (839 valid items: MIR: n=203, EIR: n=220, PIR: n=210, and FIR: n=206), frontier models reached near-ceiling accuracy on every examination, and not on medicine alone (Figure 2). Pooling under full-coverage configurations (Table 2), the strongest configuration was GPT-5.2 high (neutral) at 94.8% (795/839, 95% CI 93.0%-96.1%), with Claude Opus 4.5 (expert) at 94.3% (781/828; 11 items [EIR: n=10, and PIR: n=1] had unparsed expert-frame responses and were excluded; on the common-coverage intersection GPT-5.2 high leads 95.9% (794/828) vs 94.3% (781/828), so this does not affect the ranking). Among configurations with responses on every valid item in an examination, the maximum, selected across configurations and therefore optimistic relative to a typical model, was 99.5% (202/203) for MIR, 90.9% for (200/220) for EIR, 92.9% (195/210) for PIR, and 98.1% (202/206) for FIR.

**Figure 2.**
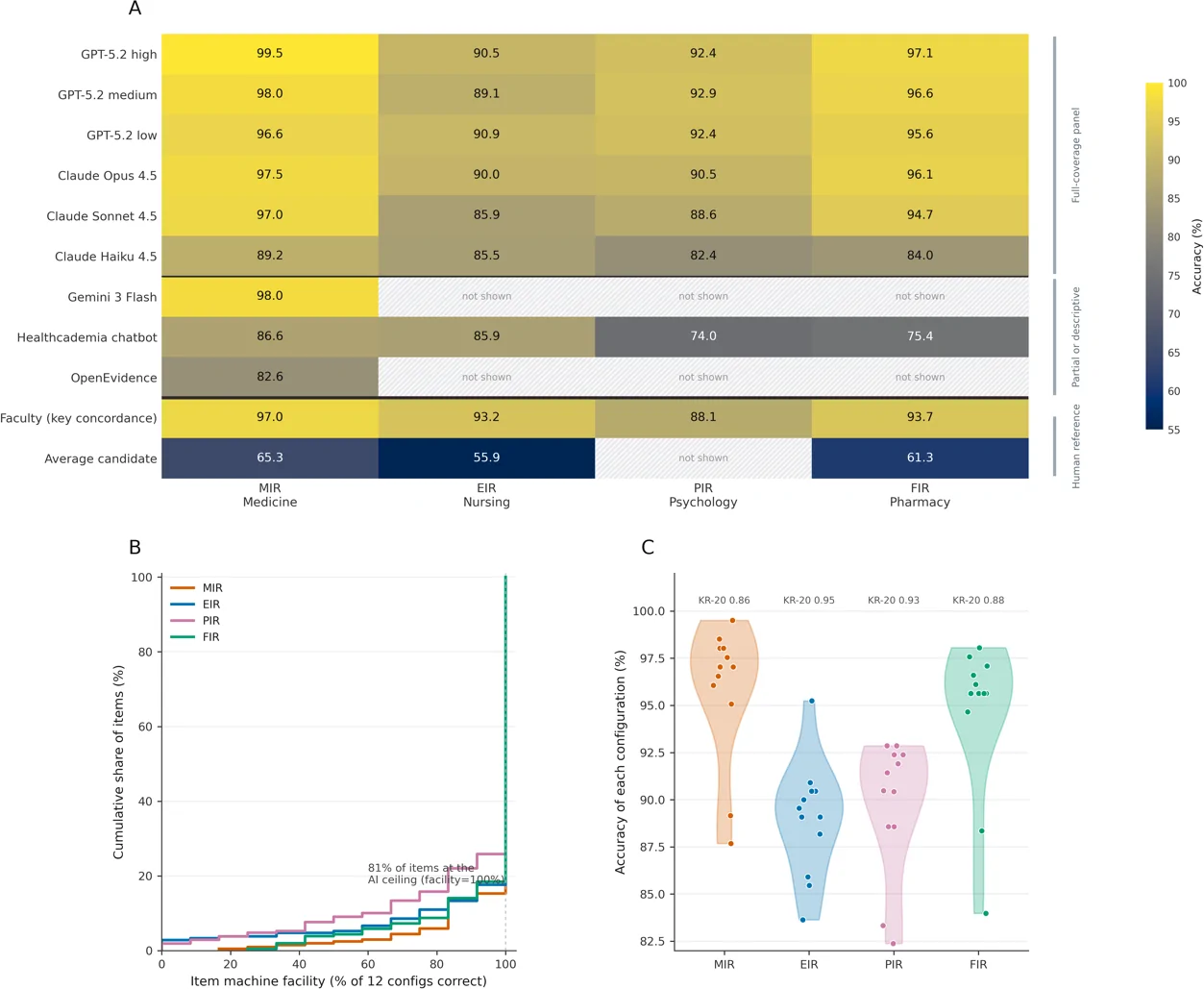
Model accuracy across the 4 examinations. (A) Per-examination accuracy for neutral-frame configurations and human reference quantities; partial or descriptive systems are separated from the main panel. Faculty agreement with the official key and candidate facility are distinct measures. (B) Distribution of machine facility; all 12 configurations answered 668 of 828 (80.7%) paired items correctly. (C) Configuration accuracies by examination, with descriptive panel KR-20. Scores cluster near the ceiling among the strongest configurations; this does not measure discrimination among human candidates.

**Table 2.** Accuracy across the four 2026 Spanish specialty-access examinations^a^.

Configuration	Pooled % (95% CI)	Correct answers (n/N)	MIR, %	EIR, %	PIR, %	FIR, %
Full-coverage frontier models: GPT-5.2						
GPT-5.2 high (neutral)	94.8 (93.0-96.1)	795/839	99.5^b^	90.5^c^	92.4^d^	97.1^e^
GPT-5.2 high (expert)	94.3 (92.5-95.7)	791/839	98.5	89.5	91.4	98.1
GPT-5.2 medium (neutral)	94.0 (92.2-95.5)	789/839	98.0	89.1	92.9	96.6
GPT-5.2 medium (expert)	94.2 (92.4-95.6)	790/839	97.0	90.5	91.9	97.6
GPT-5.2 low (neutral)	93.8 (92.0-95.2)	787/839	96.6	90.9	92.4	95.6
GPT-5.2 low (expert)	93.8 (92.0-95.2)	787/839	98.0	89.1	92.9	95.6
Full-coverage frontier models: Claude 4.5						
Claude Opus 4.5 (neutral)	93.4 (91.6-94.9)	784/839	97.5	90.0	90.5	96.1
Claude Opus 4.5 (expert)	94.3 (92.5-95.7)	781/828	96.1	95.2^f^	90.4^g^	95.6
Claude Sonnet 4.5 (neutral)	91.4 (89.3-93.1)	767/839	97.0	85.9	88.6	94.7
Claude Sonnet 4.5 (expert)	91.8 (89.7-93.5)	770/839	95.1	88.2	88.6	95.6
Claude Haiku 4.5 (neutral)	85.2 (82.7-87.5)	715/839	89.2	85.5	82.4	84.0
Claude Haiku 4.5 (expert)	85.7 (83.2-87.9)	719/839	87.7	83.6	83.3	88.3
Partial-coverage and descriptive (not in headline panel)						
Gemini 3 Flash (neutral)	—^h^	—	98.0	80.0^i^	—	92.9^j^
Healthcademia chatbot	80.4 (77.5-83.0)	645/802	86.6	85.9^k^	74.0^l^	75.4^m^
OpenEvidence (MIR only)	—	—	82.6^n^	—	—	—
Six-model ensemble (neutral)						
Majority vote	—	—	98.0	91.4	92.9	96.1
Oracle (any correct)	—	—	100.0	92.3	97.6	100.0
Items all-6-wrong (n)	—	—	0	17	5	0
Human reference						
Faculty (key-concordance), n/N (%)	—	—	97.0^o^	93.2	88.1^p^	93.7^q^
Average candidate, mean	—	—	65.3	55.9	—	61.3

a Percentages use the available responses for each listed configuration; the pooled estimate includes its numerator, denominator, and binomial 95% CI. Claude Opus 4.5 expert has 828 parsed responses (Médico Interno Residente [MIR]: n=203, Enfermero Interno Residente [EIR]: n=210, Psicólogo Interno Residente [PIR]: n=209, and Farmacéutico Interno Residente [FIR]: n=206), whereas other headline configurations have 839. Faculty agreement with the official key is not an independent reference standard. Average candidate facility includes blanks in its denominator; the difficulty analysis uses correct/answered. Maxima selected across configurations are optimistic estimates of typical performance.

b 202 out of 203.

c 199 out of 220.

d 194 out of 210.

e 200 out of 206.

f 200 out of 210.

g 189 out of 209.

h Not applicable.

i 8 out of 10.

j 13 out of 14.

k 170 out of 198.

l 151 out of 204.

m 150 out of 199.

n 142 out of 172.

o 197 out of 203.

p 185 out of 210.

q 193 out of 206.

We report 2 distinct human quantities separately. Faculty-key concordance was 97% (197/203) for MIR, 93.2% (n/N) for EIR, 88.1% (185/210) for PIR, and 93.7% (193/206) for FIR (denominators MIR 203, EIR 220, PIR 210, and FIR 206 items). Human candidate facility is reported under 2 denominators; the primary one, and the x-axis of the difficulty-transfer analysis, is correct/answered, which excludes candidate blanks and is therefore comparable to machines, which never abstain. On the 147 trap items, mean human item facility was 24.4% (SD 11.1 percentage points, 95% CI 22.7%-26.2%), whereas mean machine facility was 79.5% (SD 30.1 percentage points, 95% CI 74.7%-84.5%). Mean machine facility on the 681 nontrap items was 96.2% (SD 12.8 percentage points; 95% CI 95.2%-97.1%; Figure 3). The difficulty-transfer Spearman is essentially unchanged under either denominator (ρ=0.357 correct/answered vs ρ=0.360 correct/all; n=619, MIR, EIR, and FIR). Because faculty concordance is scored against the same official key used to score models, model-versus-faculty differences are not independent comparisons; this caveat carries into every faculty-concordance comparison below. The funder chatbot, excluded from all comparative ranking and from the first 4 validity analyses, scored pooled 80.4% (per-examination, MIR: 86.6%, EIR: 85.9%, PIR: 74.0%, and FIR: 75.4%).

**Figure 3.**
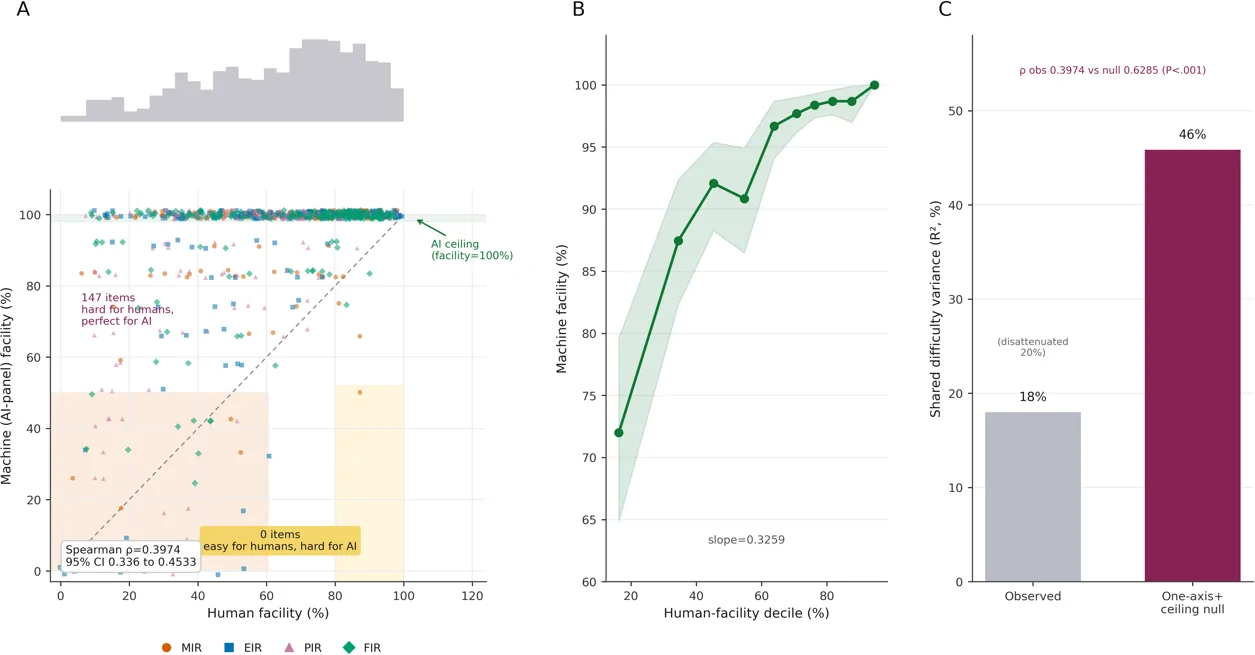
Distractor selection and surface features. (A) Machine facility on trap items (human plurality selects an incorrect option, n=147) and other items. The marker denotes mean human facility on traps (24%). (B) Dominant human-distractor selection under a uniform baseline (33%), 672 observed model errors (63.7%), and the post hoc human-plausibility baseline (64.9%); observed-minus-baseline difference –1.25 percentage points (error-event bootstrap 95% CI −4.53 to 2.19), compatible with no difference. (C) Option-level receiver operating curves for the surface-feature classifier by held-out examination (area under the curve 0.55 to 0.62). (D) Model accuracy by human facility and whether the classifier selected the correct answer. The unadjusted difference is 1.5 percentage points; the adjusted odds ratio is 0.87 (95% CI 0.72-1.04). Bands are 95% CIs. AUC: area under the curve. EIR: Enfermero Interno Residente; FIR: Farmacéutico Interno Residente; MIR: Médico Interno Residente; PIR: Psicólogo Interno Residente.

Scores clustered near the ceiling among the strongest configurations. The 6-model neutral-prompt ensemble reached majority-vote accuracies of 98% on MIR (199/203), 91.4% on EIR (201/220), 92.9% on PIR (195/210), and 96.1% on FIR (198/206). At least one ensemble member answered every MIR and FIR item correctly; all 6 missed 17 EIR items and 5 PIR items. Panel KR-20 values were 0.86 for MIR, 0.95 for EIR, 0.93 for PIR, and 0.88 for FIR. These are descriptive statistics across configurations. The observed scores offered limited separation among the strongest configurations, while weaker configurations such as Claude Haiku scored lower [23,25].

### Relationship Between Human and Machine Item Difficulty

Across 828 complete paired items, human and machine facility had a Spearman ρ of 0.40 (95% CI 0.34-0.45) and Pearson *r* of 0.42 (squared correlation approximately 0.18). Per-examination Spearman ρ values were 0.30 for MIR, 0.38 for EIR, 0.49 for PIR, and 0.38 for FIR (Table 3; Figure 4). On the 619 MIR, EIR, and FIR items with published candidate facility, the same 12-configuration panel gave correlations of 0.357 using correct/answered and 0.360 using correct/all candidates. This comparison avoids relying on reconstructed PIR facility. In the full paired corpus, 147 items had human facility below 50% (n/N) and perfect machine facility; none had human facility at least 80% (n/N) and machine facility below 50% (n/N). A further threshold comparison identified 116 items with human facility below 40% (n/N) and machine facility at least 80% (n/N).

**Table 3.** Human and machine item facility, sensitivity analyses, and simulation^a^.

Measure	Estimate	Items or observations
MIR^b^ facility: human; machine, n/N (%)	67.7; 2335/2436 (95.9)	203
EIR^c^ facility: human; machine, n/N (%)	61.7; 2340/2520 (92.9)	210
PIR^d^ facility: human; machine, n/N (%)	55.2; 2252/2508 (89.8)	209
FIR^e^ facility: human; machine, n/N (%)	65.6; 2338/2472 (94.6)	206
Pooled facility: human; machine, n/N (%)	62.5; 9265/9936 (93.2)	828
Panel KR-20:^f^ MIR; EIR; PIR; FIR	0.863; 0.953; 0.932; 0.876	12 configurations
Panel discrimination: MIR; EIR; PIR; FIR	0.57; 0.38; 0.34; 0.55	12 configurations
Pooled panel discrimination	0.45	12 configurations
MIR Spearman ρ (95% CI)	0.30 (0.17-0.42)	203
EIR Spearman ρ (95% CI)	0.38 (0.26-0.49)	210
PIR Spearman ρ (95% CI)	0.49 (0.38-0.59)	209
FIR Spearman ρ (95% CI)	0.38 (0.26-0.49)	206
Pooled Spearman ρ (95% CI)	0.40 (0.34-0.45)	828
Pearson r; squared Pearson *r*	0.42; 0.18	828
Disattenuated squared Pearson *r*	Approximately 0.20; sensitivity range 0.193 to 0.250	Assumed reliabilities
Correct under all configurations	668 (80.7%)	828
Three-examination Spearman ρ: correct/answered; correct/all candidates	0.357; 0.360	619
Mixed correct and incorrect responses: Spearman ρ	0.28	150
At least one error, including all-wrong items: Spearman ρ	0.34	160
Human facility <50%; machine facility 100%	147	828
Human facility ≥80%; machine facility <50%	0	828
Human facility <40%; machine facility ≥80%	116	828
Specified simulation: expected Spearman ρ	0.63 (95% predictive interval 0.59 to 0.66); observed 0.40; *P*<.001	2000 simulations
Specified simulation: human-hard/model-perfect count	Expected 51 (40 to 63); observed 147	2000 simulations
GLMM^g^: standardized human-facility coefficient; image coefficient	2.8368 per SD; –4.3495	9947 observations from 829 items

a Human facility is correct/answered. Machine facility is the share of 12 configurations answering correctly, primary analyses require complete coverage. PIR has human facility for 210 items and complete paired data for 209. Panel Kuder-Richardson Formula 20 and discrimination are descriptive configuration-panel statistics, not person-level test reliability. Bootstrap CIs condition on the observed single runs.

b MIR: Médico Interno Residente.

c EIR: Enfermero Interno Residente.

d PIR: Psicólogo Interno Residente.

e FIR: Farmacéutico Interno Residente.

f KR 20: Kuder-Richardson Formula 20.

g GLMM: generalized linear mixed model.

**Figure 4.**
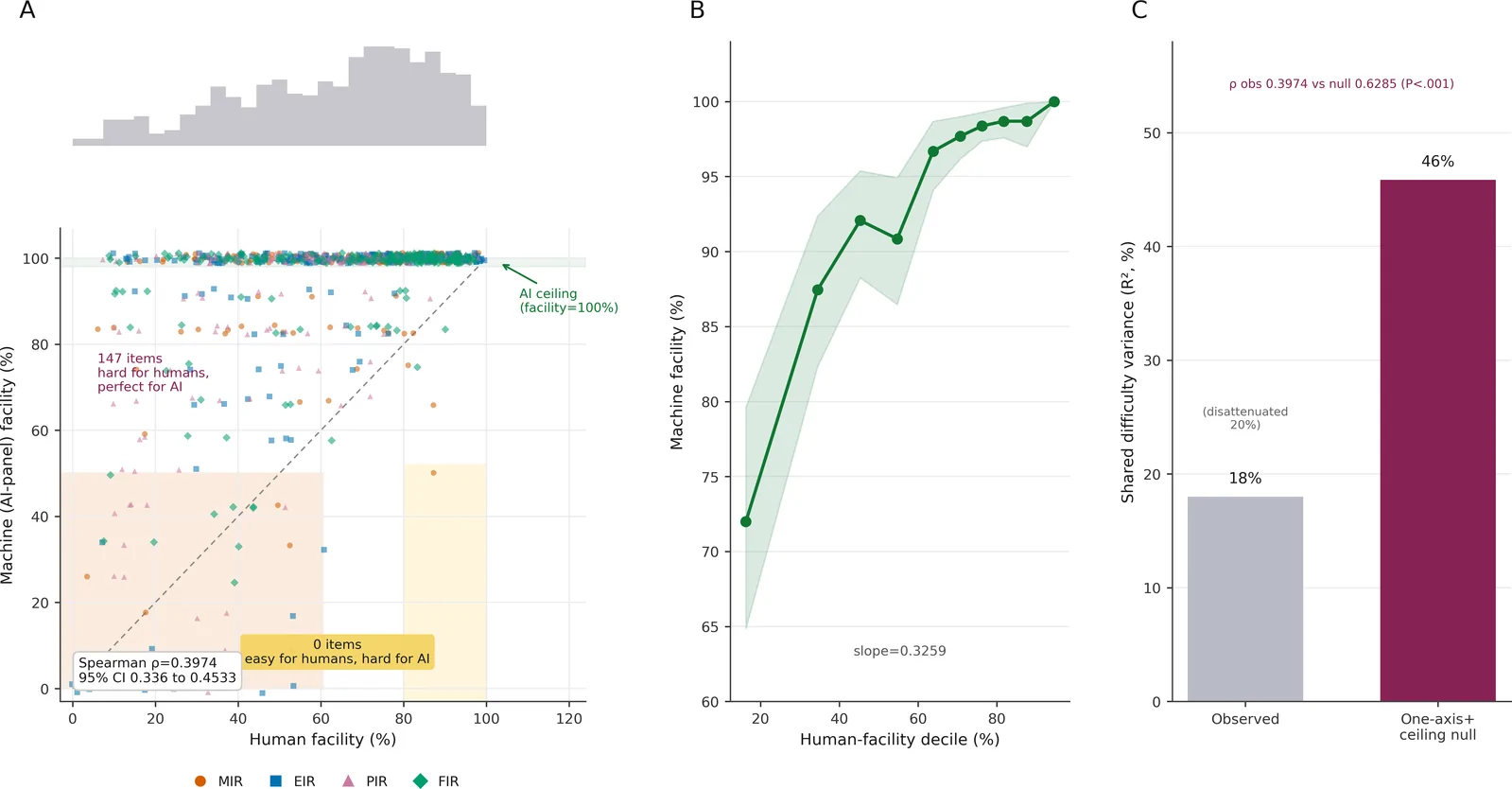
Correspondence between human and machine item facility. (A) The 828 complete paired items, with Spearman ρ=0.40 (95% CI 0.34-0.45). There were 147 items with human facility below 50% (n/N) and perfect machine facility, and none with human facility at least 80% (n/N) and machine facility below 50% (n/N). (B) Mean machine facility by human-facility decile (slope 0.33), with bootstrap 95% CIs. (C) Squared Pearson correlation (r): observed 18%, compared with 46% under the specified uniform-advantage simulation. Disattenuated shared variance is approximately 20% and varies from 19.3% to 25.0% across assumed reliabilities. The simulation result does not exclude all unidimensional models.

The asymmetric counts partly reflect the difference in overall accuracy: 668 of 828 (80.7%) paired items were at the model ceiling. Disattenuation with assumed machine and human reliabilities of 0.93 and 0.98 gave a Pearson r of 0.45 and shared variance of approximately 20% (Part S1 in Multimedia Appendix 1); these values depend on the reliability assumptions. Spearman ρ was 0.28 on the 150 items with both correct and incorrect model responses and 0.34 on all 160 items with at least one error. Mean machine facility increased from 0.72 (SD 0.34) in the hardest human-facility decile to 1.00 (SD 0.00) in the easiest (ordinary least squares [OLS] slope 0.33). The GLMM estimated coefficients of 2.84 per SD of human facility and −4.35 for the image indicator. Its 9947 observations comprised 828 items with 12 responses and one additional item with 11 responses. The small model-level random-intercept variance describes variation within this panel and does not establish equivalence between configurations.

We then tested whether the ceiling alone manufactures the weak coupling, using the ceiling-aware generative simulation (Part S1 in Multimedia Appendix 1). Under the specified one-axis-plus-uniform-advantage-plus-ceiling null, the expected Spearman ρ is 0.63 (95% predictive interval 0.59 to 0.66) and the expected hard-for-human and perfect-for-AI count is 51 (40 to 63). The observed Spearman ρ of 0.40 falls below this null (*P*<.001; 0/2000 simulations ≤ observed), and the observed 147 hard-for-human and perfect-for-AI items exceed the one-axis prediction roughly threefold. The verdict is robust across 1PL, 2PL (a=1.7), and 3PL (guessing floor c=0.25, with and without a=1.7) parameterizations and a±1-logit Δ sweep (Part S1 in Multimedia Appendix 1). By contrast, the empty reverse cell (0 easy-for-human and hard-for-AI items) is itself reproduced by the one-axis-plus-ceiling model, so the reverse-cell asymmetry is a ceiling feature and is not independent evidence of divergence. The one-axis-implied shared variance was *R*²~0.46, against the observed~0.18 (Figure 4C). The claim licensed is divergence from the specified uniform-advantage generative model, not exclusion of every unidimensional account of these data.

The observed item-difficulty correspondence was lower than this uniform-advantage model predicted. The result remains conditional on a single cohort, one run per configuration, and a coarse machine-facility measure with substantial ceiling effects. No person-level IRT or formal invariance analysis was performed, and the simulation does not establish 2 separate latent constructs.

### Sensitivity Analyses for Human-Machine Difficulty Agreement

Spearman ρ remained moderate under 7 panel definitions: 0.40 for all 12 configurations (n=828), 0.40 for neutral prompts (n=829, 6 configurations), 0.37 for expert prompts (n=828, 6 configurations), 0.39 for Claude configurations (n=828), 0.31 for GPT configurations (n=829), 0.40 for 3 Claude models plus one GPT configuration (n=829), and 0.32 for the best configuration from each vendor (n=829). The ceiling proportion ranged from 80.7% (n/N) to 92.9% (n/N). These descriptive checks show how the estimate varies with panel composition; the simulation reference of 0.63 applies to the primary panel.

Correlations were similar under alternative handling of the 11 unparsed Claude Opus 4.5 expert responses (10 EIR and 1 PIR). Among the 829 items with human facility, excluding an unparsed response from its denominator gave Spearman ρ=0.398, scoring it as incorrect gave ρ=0.398, and requiring complete model coverage gave ρ=0.397 (n=828). The 10 affected EIR items also lacked human facility, so only the PIR item changed the paired-analysis sample.

Within-examination correlations were 0.30 for MIR (95% CI 0.17-0.42; n=203), 0.38 for EIR (95% CI 0.26-0.49; n=210), 0.49 for PIR (95% CI 0.38-0.59; n=209), and 0.38 for FIR (95% CI 0.26-0.49; n=206). Fixed-effect Fisher-z pooling gave ρ=0.39 (95% CI 0.33-0.45; Q=5.10, *I*^2^=41%), close to the pooled item-level estimate of 0.40. We did not interpret the between-examination differences as established heterogeneity given the single-run design.

Across the assumed human-reliability range of 0.90 to 0.98 and machine-reliability range of 0.80 to 0.95, disattenuated shared variance was 19.3% to 25.0%. This is an assumption-dependent sensitivity analysis: human reliability was not estimated from these data, and panel KR-20 is not conventional human test reliability.

### Error and Distractor Concordance Between Models and Humans

We identified 147 trap items (147/828, 17.8% of the paired corpus; per-examination: MIR: n=22, EIR: n=35, PIR: n=54, and FIR: n=36) on which the human plurality selected a wrong option. On trap items, human accuracy was 24.4% (95% CI 22.7%-26.2%) versus AI 79.5% (95% CI 74.7-84.5), against nontrap 96.2% (95% CI 95.2-97.1; Figure 3). Models therefore answered many items correctly even when the modal human answer was incorrect.

The 147 trap items and the 147 items hard for humans but perfect for the AI panel overlapped in 77 items (phi=0.42). There were 70 trap-only items, 70 hard-for-human and perfect-for-AI-only items, and 611 items in neither group. Mean human and machine facility were respectively 0.28 (SD 0.10) and 1.00 (SD 0.00) in the overlap, 0.21 (SD 0.11) and 0.57 (SD 0.31) in the trap-only group, 0.43 (SD 0.05) and 1.00 (SD 0.00) in the hard-for-human and perfect-for-AI-only group, and 0.74 (SD 0.14) and 0.96 (SD 0.13) in the remaining items. The definitions therefore identify related but distinct item sets.

Across 672 incorrect responses from the declared 12-configuration panel, models selected the dominant human distractor with probability 0.6369, compared with 0.6494 under the post hoc plausibility-weighted baseline. The difference was −1.25 percentage points (error-event bootstrap 95% CI −4.53 to 2.19), compatible with no difference. This does not establish either shared misconceptions or systematic divergence beyond option plausibility. The probability of selecting the human modal option was 0.3661. The mean within-item Pearson *r* between human and model distributions over incorrect options was 0.50 (SD 0.61) across 161 items with defined correlations. Human response entropy and model confidence had a descriptive Spearman ρ of −0.30 (n=9851); mean confidence was 93.57 (SD 10.01) at entropy below 0.3 and 87.37 (SD 13.28) at entropy above 0.7.

### Surface Features and Correct-Answer Prediction

A surface-feature classifier with no medical knowledge, cross-validated leave-one-examination-out, recovered the keyed answer at 34.4% (289/839, 95% CI 31.3-37.7) against a 25% chance baseline (Figure 3). The signal is, however, almost entirely a single item-writing artifact: a longest-option heuristic applied alone, with no classifier at all, recovers the key at 34.6% (290/839), matching or exceeding the classifier. The same holds per examination, where classifier and longest-option accuracies are near-identical (MIR n, 39.9% vs n, 39.4%; EIR n, 32.7% vs n, 32.7%; PIR n, 32.4% vs n, 31.9%; FIR n, 33.0% vs n, 34.5%; denominators MIR 203, EIR 220, PIR 210, and FIR 206). We therefore describe this narrowly, as a length-dominated item-writing artifact rather than a broad surface-leakage construct: the classifier’s leading coefficient is option length (len_z=0.287; longer options more often correct), with weaker cues (has_absolute=−0.509, is_allnone=0.47, and stem_overlap=−0.34; Part S1 in Multimedia Appendix 1), and a within-examination cross-validated solvability was comparable, indicating the signal is item-format-intrinsic.

Mean AI accuracy was 93.2% (SD) on items solved by the surface-feature classifier and 91.7% (SD) on the remaining items (unrounded difference 1.6 percentage points); corresponding mean human facility was 66% (SD) and 61% (SD). After adjustment for human facility, the coefficient was -0.145 (95% CI −0.325 to 0.035; *P*=.11), equivalent to an odds ratio (OR) of 0.87 (95% CI 0.72-1.04). We found no clear evidence of differential reliance on the measured cues, but this interval does not establish absence of an effect. The probe addresses a limited set of surface features [28,29] and cannot determine whether high scores reflect prior exposure to related content. Controlled item rewrites remain a planned follow-up.

### Exploratory Audit of Errors and Model Explanations

The exploratory audit assessed 253 incorrect frontier-model responses (Figure 5 and Table 4). The Opus judge labeled 99 as knowledge gaps (n=_, 39.1%, bootstrap 95% CI 34-45), 68 as reasoning errors (n=_, 26.9%), 36 as fine-distractor errors (n=_, 14.2%), 21 as visually dependent (n=_, 8.3%), 13 as vignette misreads (n=_, 5.1%), 12 as item flaws or keying issues (n=_, 4.7%), and 4 as hallucinations (n=_, 1.6%). No response received the primary Spanish-language label (0/253), which does not exclude subtler language effects. The sample contained more Claude and weaker-model errors; GPT-5.2 high errors were more often labeled reasoning or fine-distractor errors (Part S1 in Multimedia Appendix 1). All labels concern generated explanations and remained unvalidated by clinicians.

**Figure 5.**
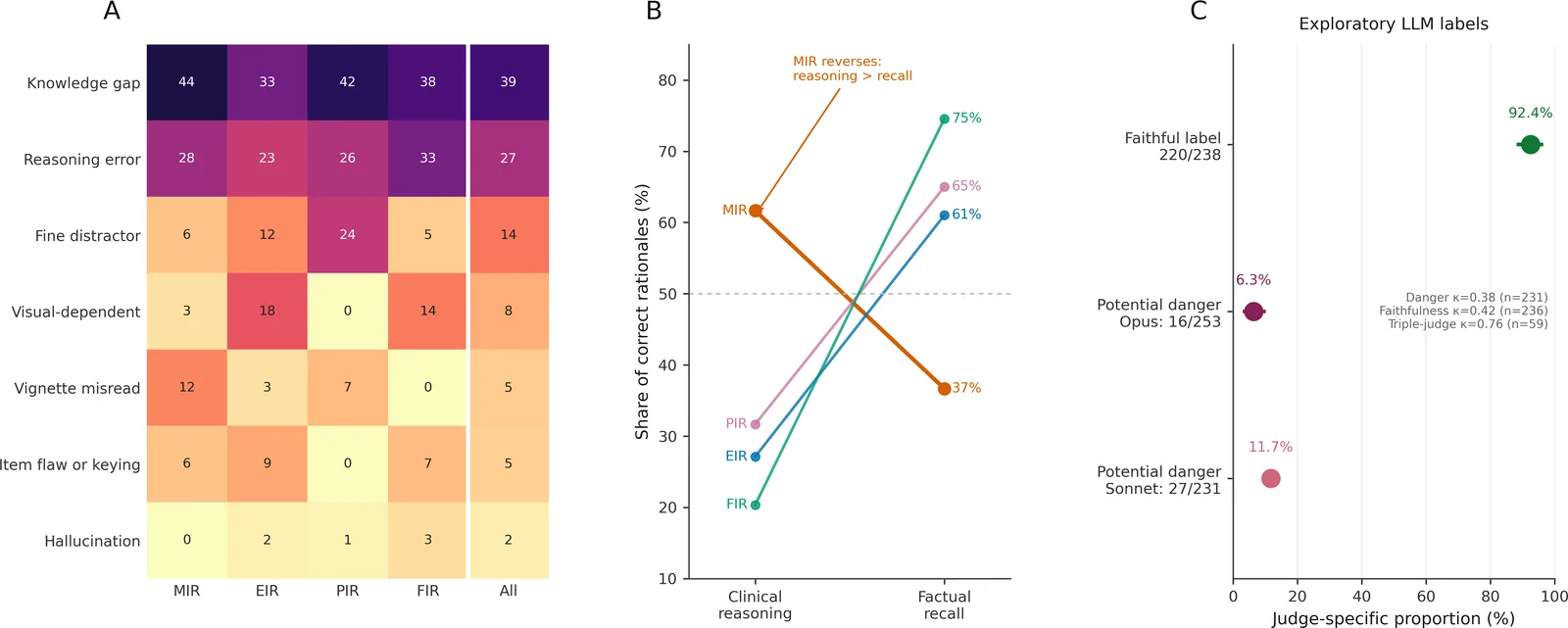
Exploratory large language model–judge audit of generated explanations. (A) Primary failure labels among 253 incorrect responses, by examination. (B) Expressed reasoning labels for 238 correct GPT-5.2 high responses; clinical reasoning exceeds recall on Médico Interno Residente (MIR). (C) Faithfulness and potential-danger labels, with bootstrap 95% CIs where shown. Opus-Sonnet agreement uses 231 jointly labeled errors (failure κ=0.43 and danger κ=0.38) and 236 jointly labeled correct rationales (reasoning κ=0.65 and faithfulness κ=0.42). The separate triple-judge estimate uses 59 MIR and Enfermero Interno Residente (EIR) errors (Fleiss κ=0.76). Psicólogo Interno Residente (PIR) and Farmacéutico Interno Residente (FIR) lack triple-judge coverage, but are included in the 2-judge audit. Labels have not been validated by clinicians and do not measure internal computation or clinical risk. LLM: large language model.

**Table 4.** Exploratory large language model-judge audit. Labels describe generated explanations and have not been validated by clinicians^a^.

Label or measure	Overall, n/N (%)	MIR, n/N (%)	EIR, n/N (%)	PIR, n/N (%)	FIR, n/N (%)
Knowledge gap	99/253 (39.1)	14/32 (44)	22/66 (33)	41/97 (42)	22/58 (38)
Reasoning error	68/253 (26.9)	9/32 (28)	15/66 (23)	25/97 (26)	19/58 (33)
Fine distractor	36/253 (14.2)	2/32 (6)	8/66 (12)	23/97 (24)	3/58 (5)
Visual dependence	21/253 (8.3)	1/32 (3)	12/66 (18)	0/97 (0)	8/58 (14)
Vignette misread	13/253 (5.1)	4/32 (12)	2/66 (3)	7/97 (7)	0/58 (0)
Item flaw or keying	12/253 (4.7)	2/32 (6)	6/66 (9)	0/97 (0)	4/58 (7)
Hallucination	4/253 (1.6)	0/32 (0)	1/66 (2)	1/97 (1)	2/58 (3)
Spanish-language error	0/253 (0.0)	0/32 (0)	0/66 (0)	0/97 (0)	0/58 (0)
Faithful explanation	220/238 (92.4)	58/60 (97)	50/59 (85)	56/60 (93)	56/59 (95)
Factual recall	141/238 (59.2)	22/60 (37)	36/59 (61)	39/60 (65)	44/59 (75)
Clinical reasoning	84/238 (35.3)	37/60 (62)	16/59 (27)	19/60 (32)	12/59 (20)
Elimination	12/238 (5)	0/60 (0)	7/59 (12)	2/60 (3)	3/59 (5)
Test heuristic	1/238 (0.4)	1/60 (2)	0/59 (0)	0/60 (0)	0/59 (0)
Potential danger					
Opus	16/253 (6.3)	—^b^	—	—	—
Sonnet	27/231 (11.7%)	—	—	—	—
Triple-judge Fleiss κ (n=59)	0.76	MIR and EIR subset	—	Not assessed	Not assessed
Opus-Sonnet					
Failure, κ (n=231)	0.43	All 4 examinations	—	—	—
Danger, κ (n=231)	0.38	All 4 examinations	—	—	—
Faithfulness, κ (n=236)	0.42	All 4 examinations	—	—	—
Reasoning, κ (n=236)	0.65	All 4 examinations	—	—	—

a Opus labeled 253 errors and 238 correct-answer rationales. Sonnet comparisons use 231 jointly labeled errors and 236 jointly labeled correct rationales. Triple-judge assessment was limited to 59 MIR and EIR errors. Counts and percentages refer to the available labels within each sample; CIs are bootstrap 95% where shown.

b Not applicable.

Opus and Sonnet labels showed Cohen κ=0.43 for failure mode and κ=0.38 for potential danger on 231 jointly labeled errors, and κ=0.65 for reasoning mode and κ=0.42 for faithfulness on 236 jointly labeled correct-answer rationales. Opus flagged 16 of 253 errors as potentially dangerous (6.3%, bootstrap 95% CI 3.6-9.5); Sonnet flagged 27 of 231 (11.7%). These are judge-specific estimates with different available-label denominators. Their difference and the limited agreement show uncertainty in the audit; neither estimate is a clinical risk rate or a lower bound on harm. Profession-matched clinician review of flagged errors and a control sample, together with a cross-vendor judge, is needed before any safety interpretation.

Among 238 correct GPT-5.2 high responses, the Opus judge classified 220 rationales as faithful (92.4%, 95% CI 88.7-95.8), 15 as partially faithful (6.3%), 2 as unfaithful but correct (0.8%), and 1 as unverifiable (0.4%). A total of 2 contained a false statement (n=238, 0.8%). Expressed reasoning was classified as factual recall in 141 (59.2%, 95% CI 53.4-65.6), clinical reasoning in 84 of 238 (35.3%), elimination in 12 of 238 (5%), and a test heuristic in 1 of 238 (0.4%). Clinical reasoning exceeded recall on MIR; the reverse held on EIR, PIR, and FIR. These labels describe one model’s explanations and do not verify its internal reasoning process.

### Certainty, Latency, Image Availability, and Prompt Frames

Stated confidence distinguished correct from incorrect responses within the panel (GPT-5.2 AUROC approximately 0.87 to 0.90). The highest AUROC was 0.90 for GPT-5.2 high with the expert prompt, with a 27.6-point difference in mean confidence between correct and incorrect answers. Ten of 12 configurations were net underconfident, the 2 Claude Haiku configurations were net overconfident. ECE ranged from 0.030 to 0.075 within the declared panel (Figure 6A). These discrimination and calibration measures do not establish that confidence can safely guide decisions. Higher GPT-5.2 reasoning effort increased observed latency, but single runs cannot establish a difference in expected accuracy. Claude Haiku had the shortest median latency, approximately 1.56 (IQR 1.34-1.87) seconds under the neutral prompt and 1.61 (IQR 1.42-1.94) seconds under the expert prompt, with 781 recorded responses in each configuration (Figure 6B).

**Figure 6.**
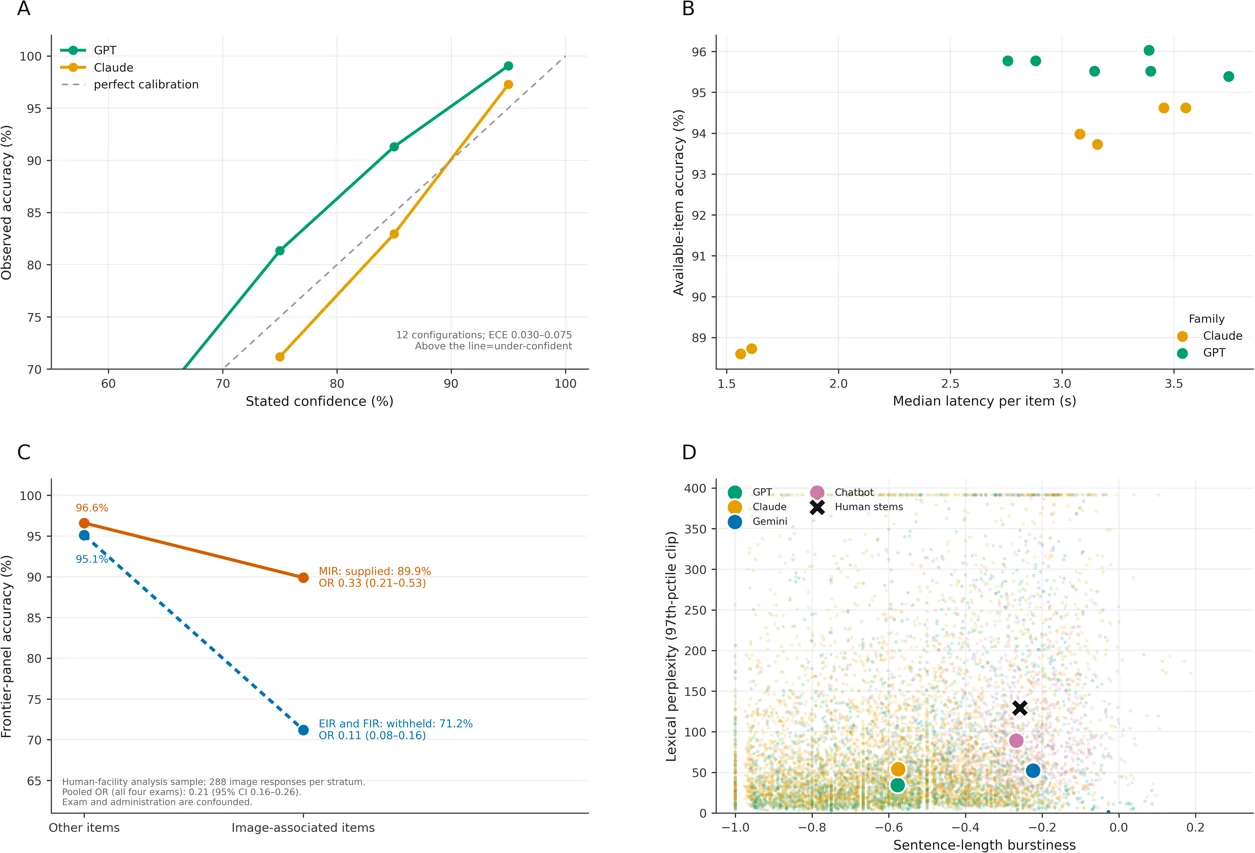
Calibration, latency, image administration, and stylometry. (A) Accuracy against stated confidence; 10 of 12 configurations were net underconfident despite confidence discriminating correct and incorrect answers. (B) Pooled accuracy against median response latency; single runs do not establish differences in expected accuracy. (C) Image-associated and other item accuracy. Médico Interno Residente (MIR) images were supplied (89.9% vs 96.6%; adjusted odds ratio [OR] 0.33, 95% CI 0.21-0.53); Enfermero Interno Residente (EIR) and Farmacéutico Interno Residente (FIR) images were withheld (71.2% vs 95.1%; OR 0.11, 95% CI 0.08-0.16). The pooled logistic OR across all 4 examinations (9947 observations), 0.21 (95% CI 0.16-0.26) differs from the conditional generalized linear mixed models coefficient of –4.35. Examination and administration differences prevent a causal interpretation. (D) Style measures by model family; classifier accuracy was 82.8%, compared with a 56.1% majority-class baseline. This does not identify official items as AI-written. Panel (C) uses the human-facility-observed analysis sample (288 image-response observations in each image-availability stratum).

The association between image items and accuracy differed by administration route (Figure 6C). On MIR, where images were supplied, accuracy was 89.9% on image items and 96.6% on other items (human facility–adjusted OR 0.33, 95% CI 0.21-0.53). On EIR and FIR, where images were withheld, the corresponding values were 71.2% and 95.1% (OR 0.11, 95% CI 0.08-0.16). Across all 4 examinations (9947 observations), the pooled logistic estimate was OR 0.21 (95% CI 0.16-0.26). It combines differences in examinations, visual input, and administration; it cannot isolate visual reasoning ability. The GLMM coefficient of –4.35 is a separate conditional estimate from a model with item and model random intercepts, and is not the logarithm of the pooled logistic odds ratio. These regression analyses used items with human facility, comprising 24 MIR image items and 24 EIR and FIR image items (10 EIR and 14 FIR), with 288 image-response observations in each stratum.

The panel correlation was 0.40 under neutral prompts and under expert prompts or 0.37, the statistic is Spearman rank correlation coefficient. The mean absolute change in model accuracy between frames was 0.4 percentage points (maximum 0.9). These comparisons assess sensitivity to prompt framing, not stochastic variability under a fixed prompt. Repeated queries across seeds and temperatures were not available for this secondary analysis (Part S1 in Multimedia Appendix 1).

### Writing Style of Model Explanations (Supporting Analysis)

A random-forest classifier identified the generating family from style alone at 82.8% (n/N) on the deduplicated classifier sample (n=8183), against the appropriate majority-class baseline of 56.1% (n/N), the signal carried substantially by output length and formatting (Figure 6D). Given the circularity of perplexity- and burstiness-based heuristics popularized for AI detection [48,49] and a Gemini content confound, we present this as supporting only and *not* as a claim that any official item is AI-written; the full analysis is in Part S1 in Multimedia Appendix 1.

## Discussion

### Principal Findings

Across 839 items from 4 Spanish health-professions examinations, the strongest model configuration answered 795 (94.8%) correctly. All 12 configurations answered 668 of 828 (80.7%) paired items correctly, leaving limited score separation among the strongest configurations. Human and machine item facility showed moderate correspondence (Spearman ρ=0.40, 95% CI 0.34-0.45). Thus, high overall model accuracy did not reproduce the human ordering of item difficulty.

The correlation remained moderate across alternative panels, scoring rules, and examination-stratified pooling. It was also lower than predicted by the specified simulation with a shared difficulty axis and uniform model advantage. This supports a difference in the observed response patterns, but does not exclude all unidimensional models or establish formal measurement noninvariance. After restricting the distractor analysis to the same declared panel, selection of the dominant human distractor was compatible with the post hoc plausibility-weighted baseline. That analysis therefore provides no clear additional evidence of divergence beyond option plausibility. The surface-feature analysis found no clear evidence that the measured cues explained model performance after adjustment for human facility.

Kane framework separates accurate scoring from generalization, extrapolation to professional competence, and decisions based on a score [17-20]. Our data support exact-match scoring and describe performance on these items, while leaving generalization across occasions and test forms uncertain (Table 1). They do not establish extrapolation to clinical performance for models or candidates. Human validation evidence cannot simply be assumed to apply to model respondents [23-26]. The educational implication is therefore to assess the meaning of a model’s score before using it as evidence of professional competence.

Prior exposure remains an alternative explanation for high accuracy [25,33]. Public release after the examinations does not establish that related questions or concepts were absent from model training, and the surface-feature probe is not a contamination test. Controlled paraphrases and repeated model queries would help assess response sensitivity, although neither alone would establish the absence of training-data overlap. These analyses were not performed here.

For educators using LLMs to review item banks, machine and human facility should be reported separately. An item readily answered by a model can still be difficult for candidates. We did not measure human item discrimination and therefore cannot conclude that such items have lost their value for ranking candidates. The trap-item results identify response patterns for further investigation; they do not establish that a particular distractor design improves assessment.

The rationale audit provides exploratory descriptions rather than evidence of internal computation. Correct GPT-5.2 high explanations more often received a recall label overall, while clinical-reasoning labels predominated on MIR. A judge’s assessment of faithfulness cannot verify that a generated explanation reflects the process that produced the answer. Potential-danger labels also varied between the same-family judges and lacked clinician validation. These findings motivate profession-matched adjudication and cross-vendor replication, rather than a safety estimate or an educational recommendation based on the danger percentage.

### Comparison With Prior Work

The study adds item-level evidence to concerns about the construct validity and saturation of AI benchmarks [23-26], extending previous MIR accuracy studies [11-15] across 4 professions. The length-based probe shows a narrow item-writing artifact, but the uncertain adjusted association with model correctness does not settle the broader shortcut-learning question [27-34]. Our exploratory explanation labels relate to work distinguishing knowledge, reasoning, metacognition, and explanation faithfulness [35-38]. Stylometry likewise relates to model-text detection research [48,49], whose limitations and potential biases remain relevant [50,51]. As LLMs are used to draft examination items [52,53], provenance and secure authoring warrant attention [54]; our stylometric results do not identify any official item as AI-written.

Educational scaffolding offers a rationale for supervised formative uses of LLMs [4-7], but examination accuracy alone does not establish their benefit for learning. Such uses need evaluation of feedback quality, learner outcomes, overreliance, and equitable access, alongside the concerns described in prior reviews [1-3]. Similarly, better model scores do not demonstrate a solution to workforce distribution or retention [41]. Image-rich or station-based assessment may broaden the skills sampled, but superiority to the present examinations was not tested here.

### Limitations

This was a single [14] cohort using four examinations with a shared format. Generalization to other languages, formats, years, and health systems is untested. Training-data overlap, including recurrent concepts and prior questions, was not assessed; the surface-feature probe cannot exclude contamination.

Each configuration was queried once. Item-bootstrap CIs are conditional on those responses and omit run-to-run uncertainty; changing prompt frames does not estimate that uncertainty. Machine facility is a coarse proportion over related configurations, with substantial ceiling effects. Panel KR-20 is descriptive, and disattenuation depends on assumed reliabilities. The simulation tests a specified uniform-advantage model and cannot rule out every unidimensional account. Human facility derives from aggregate responses, reconstructed for PIR; no person-level item response theory (IRT), human item-discrimination analysis, or formal DIF test was possible. Candidate facility and faculty agreement with the official key are distinct comparisons.

All analyses were exploratory, *P* values were unadjusted, and the plausibility-weighted distractor baseline was constructed post hoc. Its error-event bootstrap does not account for dependence among responses to the same item or from related configurations. Surface features were limited and largely captured option length; an uncertain adjusted association does not establish absence of shortcut use. The rationale audit used same-family LLM judges, with different label coverage, no profession-matched clinician validation, and a physician-oriented rubric across 4 professions. Correct-answer analyses covered GPT-5.2 high only, with reasoning labels that varied by examination. Neither explanation labels nor potential-danger flags establish internal mechanisms or clinical safety. Stylometry is confounded by genre, output length, and deployment.

Image effects are confounded with examination and administration: MIR images were supplied manually, whereas EIR and FIR images were withheld. These contrasts do not isolate visual competence or establish a benefit of image-rich assessment. More broadly, the study measured MCQ responses, not learning outcomes, clinical performance, or the validity of these examinations for human candidates [18-20,26]. Controlled rewrites, repeated queries, and profession-matched adjudication remain planned follow-up work.

### Conclusions

In the January 2026 MIR, EIR, PIR, and FIR examinations, LLMs achieved high accuracy, with limited score separation among the strongest configurations and only moderate correspondence with human item difficulty. These findings caution against treating high model scores as equivalent evidence of human professional competence. They do not establish clinical performance or invalidate the examinations for candidates. The explanation audit and proposed changes to assessment remain exploratory. Further validation requires repeated model queries, evaluation of possible contamination, and profession-matched review before examination scores inform claims about clinical or educational use.

## Supplementary material

10.2196/106199 Multimedia Appendix 1 Methods, analyses, results tables, and the rubric for auditing model-generated explanations.

10.2196/106199 Multimedia Appendix 2 Code and reproduction instructions for analyses restricted to the declared 12-configuration model panel.

## Abbreviations

AUROC: area under the receiver operating characteristic curve
DIF: differential item functioning
ECE: expected calibration error
EIR: Enfermero Interno Residente
FIR: Farmacéutico Interno Residente
FSE: Formación Sanitaria Especializada
GenAI: generative AI
GLMM: generalized linear mixed model
IRT: item response theory
KR-20: Kuder-Richardson formula 20
LLM: large language model
MCQ: multiple-choice question
MIR: Médico Interno Residente
OLS: ordinary least squares
OR: odds ratio
PIR: Psicólogo Interno Residente
USMLE: United States Medical Licensing Examination
