# Supplementary material for: Construct Validity of Frontier Large Language Models on 4 Spanish Health Professions Specialty-Access Examinations (MIR, EIR, PIR, and FIR): Cross-Sectional Benchmarking and Psychometric Study
Source: JMIR Med Educ. 2026 Oct 29;12:e106199. doi: 10.2196/106199 (PMC13718280; doi:10.2196/106199)
Supplement: Supplementary file 1 [file mededu-v12-e106199-s001.docx]

# **Multimedia Appendix**

# **Part S1. Supplementary Methods and Robustness Analyses**

## M1. Pre-specification status of each pillar

All five analytic pillars were designated exploratory and hypothesis-generating; accuracy against the official key is reported as supporting context only. The pre-specification status of each pillar, including post-hoc deviations adopted as primary analyses, is recorded here in full.

- Pillar 1 (difficulty transfer). Pre-specified as a two-axis rank-association analysis. The disattenuation correction, the stratified machine-facility-by-human-difficulty description, the generalized linear mixed model (GLMM), and the ceiling-aware generative simulation were added during analysis to address the ceiling confound raised in review; they operate on the existing item-level data and introduce no new measurements. The simulation replaces what an earlier draft had designated a "decisive future analysis."
- Pillar 2 (misconception sharing). The comparison of error concordance against the uniform-among-wrong baseline (0.33) was pre-specified. The plausibility-weighted null was added post hoc and adopted as the primary comparison for error concordance. It was not pre-specified. The uncertainty-alignment correlation is reported descriptively only, with inferential language removed.
- Pillar 3 (surface leakage). The leave-one-exam-out cross-exam solvability estimate was pre-specified. The within-exam cross-validated solvability, the modal-position and length-only baselines, and the human-facility-adjusted exploitation contrast were added to address the alternative that cross-exam transfer reflects shared format rather than item-intrinsic leakage.
- Pillar 4 (audit). Designated provisional and exploratory throughout. The triple-judge Fleiss κ subset and the two-judge Opus-versus-Sonnet Cohen κ over the available paired records are reported as two distinct reliability regimes that are never conflated. Human-clinician adjudication and a cross-vendor (non-Claude) judge are stated prerequisites for any safety claim, not downstream next steps.
- Pillar 5 (stylometry). Exploratory and supporting only; a single summary panel appears in the main text, with the full analysis here. It is explicitly not a claim that any official examination item is machine-written.

## M2. Pillar 1: Ceiling-aware generative simulation: full mechanics and sensitivity

### M2.1 Rationale and design

The human-machine difficulty correlation is range-restricted because 80.7% of items sit at the artificial-intelligence (AI) ceiling (panel all-correct) and machine facility is a coarse proportion over a 12-configuration panel (per-item standard error up to ≈0.15). When one population vastly out-scores the difficulty range, the rank correlation is mechanically compressed even under a single shared latent demand. We used a ceiling-aware generative simulation to assess whether the observed weak coupling was compatible with a specified shared-difficulty model with a uniform AI advantage.

We specified a one-parameter logistic (1PL) generative null in which a single shared latent item difficulty, calibrated to the empirical human facility, is viewed by the AI panel through a uniform AI ability advantage of Δ logits (the only group difference), then pushed through the 12-configuration AI panel and censored at the empirical ceiling. This one-axis-plus-ceiling model allows a uniform performance advantage but no item-specific divergence in difficulty; conclusions are conditional on that model specification.

### M2.2 Non-circular calibration of Δ

Δ was calibrated so that the simulated mean machine facility equals the *observed mean machine facility* (not the observed ρ). The test is therefore not circular: the null is fitted to the *level* of machine performance, not to the human-machine coupling it is used to evaluate. For the 1PL specification the solved value was Δ = 2.656 logits.

### M2.3 Procedure

Over 2000 simulations we recorded the null predictive distribution of the human-machine Spearman ρ and of the extreme-cell counts (hard-for-human/perfect-for-AI; easy-for-human/hard-for-AI), and located the observed values within them. Because this null contains exactly one latent difficulty axis, an observed coupling within its predictive interval would be compatible with that specification, whereas coupling below it would indicate lack of fit to the specified uniform-advantage model.

### M2.4 Primary result

Under the 1PL one-axis-plus-ceiling null the expected Spearman is 0.63 (95% predictive interval 0.59 to 0.66) and the expected hard-for-human/perfect-for-AI count is 51 (40 to 63). The observed Spearman of 0.40 falls *below* this null (*P*<.001; 0/2000 simulations ≤ observed), and the observed 147 hard-for-human/perfect-for-AI items exceed the one-axis prediction roughly threefold. The one-axis-implied shared variance was R²≈0.46, against the observed ≈0.18. The empty reverse cell (0 easy-for-human/hard-for-AI items) is itself reproduced by the one-axis-plus-ceiling model, so the reverse-cell asymmetry is a ceiling/uniform-advantage feature and is *not* independent evidence of divergence.

### M2.5 Sensitivity: parameterization (1PL/2PL/3PL) and Δ sweep

As a sensitivity analysis we re-ran the null under 2PL (discrimination a=1.7) and 3PL (guessing floor c=0.25, with and without a=1.7) parameterizations and across a ±1-logit sweep of Δ. The observed-below-null verdict holds throughout.

| **Parameterization** | **One-axis null ρ (95% predictive interval)** | **Observed ρ below null?** |
| --- | --- | --- |
| 1PL | 0.6282 (0.5904 to 0.6656) | Yes |
| 2PL (a=1.7) | 0.6278 (0.5917 to 0.6652) | Yes |
| 3PL (c=0.25) | 0.6309 (0.6011 to 0.6603) | Yes |
| 3PL (c=0.25, a=1.7) | 0.6319 (0.6004 to 0.6636) | Yes |

Across the ±1-logit Δ sweep (1PL), the observed ρ remained below the null at every value of Δ. The observed ρ stays below the one-axis-plus-ceiling null across 1PL/2PL/3PL and the Δ sweep, indicating divergence from the tested uniform-advantage specifications.

### M2.6 Caveats

This is a single-run, item-level simulation: the AI-panel difficulty remains a coarse, ceiling-censored proxy; no person-level item response theory (IRT) was fitted; the empty reverse cell is a ceiling feature, not extra evidence; and we do not claim two fully orthogonal constructs. Repeated model runs and person-level data would be needed to assess broader measurement-invariance claims.

## M3. Pillar 1: Disattenuation arithmetic

Classical attenuation theory corrects an observed correlation for the unreliability of each measured axis. The disattenuated correlation is r_xy / √(r_xx · r_yy), where r_xy is the observed Pearson correlation and r_xx, r_yy are the reliabilities of the two axes.

- Observed Pearson r_xy = 0.4243 (observed descriptive shared variance R² ≈ 0.18).
- Machine-facility reliability r_yy = 0.9265 (estimated over the 12-configuration panel).
- Assumed human-facility reliability r_xx = 0.98. This value was not estimated from person-level responses; the sensitivity grid in Table S15 evaluates alternative reliability assumptions.

Disattenuated Pearson = 0.4243 / √(0.98 × 0.9265) = 0.4453, giving disattenuated shared variance = 0.4453² ≈ 0.1983 (≈20%).

Under these assumed reliabilities, the corrected association remains modest; this sensitivity analysis does not exclude measurement error or establish separate constructs. We report the disattenuated ≈20% alongside the observed descriptive ≈18% consistently, and we do not use variance-explained as the primary framing. As a complementary robustness check, on the 150 mixed-response items (at least one configuration correct and at least one incorrect; a subset of the 160 items with at least one error) the coupling remained weak at Spearman 0.2756 (P=6.4×10⁻⁴), a descriptive sensitivity result that remains conditional on the observed panel.

## M4. Pillar 2: Plausibility-weighted-null derivation

The human top distractor is, by construction, usually the dominant wrong option, so any informed eliminator tends to converge on it; the uniform-among-wrong baseline of 0.33 is therefore not the appropriate null. We derived a plausibility-weighted null: the probability that an informed respondent who discards implausible options would land on the human top distractor, obtained by weighting the residual wrong options by their human-choice share. For each item the three wrong options are weighted by the proportion of human candidates selecting them, and the probability mass on the dominant (human top) distractor under that weighting gives the per-item plausibility-weighted null; these probabilities are then averaged over eligible model-error events. The reported confidence interval for the observed-minus-null contrast resamples error events; repeated events from the same item or configuration are not clustered, so this interval is conditional and may understate uncertainty.

- Observed P(error → human top distractor) = 0.6369 across 672 eligible error events from the 12-configuration primary panel (uniform null 0.3333).
- Plausibility-weighted null = 0.6494.
- Excess over plausibility-weighted null = −0.0125 (−1.25 percentage points; event-bootstrap 95% CI −0.0453 to +0.0219, or −4.53 to +2.19 percentage points).

The confidence interval includes zero. The analysis therefore provides no clear evidence that primary-panel errors converge on, or diverge from, the dominant human distractor beyond the human-choice-weighted reference distribution. It does not identify the models' internal elimination process or establish shared or distinct latent constructs. The mean per-item Pearson correlation between human and model wrong-option distributions was 0.4998 across 161 eligible items; this plausibility-confounded quantity is descriptive. Model-error rows with malformed multi-option strings were excluded from the concordance denominator. These corrected estimates use only the declared 12 configurations; the previously reported values inadvertently included descriptive systems. The estimator and bootstrap procedure were retained, with the model filter corrected.

For completeness, per-exam trap facilities (AI panel) were: MIR AI-trap 0.8523, AI-non-trap 0.9715, human-trap 0.243 (n=22); EIR 0.8024 / 0.9538 / 0.239 (n=35); PIR 0.733 / 0.9554 / 0.2412 (n=54); FIR 0.8472 / 0.9667 / 0.2532 (n=36). The 147 trap items and the 147 hard-for-human/perfect-for-AI items overlap in 77 items (52%; union 217); they are neither disjoint nor co-extensive.

## M5. Pillar 3: Surface-feature ("no-knowledge") solvability: feature details

To estimate construct-irrelevant variance from surface cues, the multiple-choice-question (MCQ) analogue of "Clever Hans" shortcut learning, we asked how far each exam is solvable from option *surface features alone*, with the stem's medical content withheld. For every option we computed format-only features:

- character length and word length;
- longest-option and shortest-option flags (within the item);
- within-item length z-score (len_z);
- option position;
- presence of absolute terms (has_absolute);
- presence of negation;
- presence of numerals;
- "all of the above" / "none of the above" patterns (is_allnone);
- lexical overlap with the stem (stem_overlap).

An L2-regularized logistic classifier predicted the correct option under a leave-one-exam-out scheme, with item-level accuracy reported overall and per exam with bootstrap 95% CIs (n=839). As a complement to the cross-exam transfer estimate (which could reflect shared format rather than item-intrinsic leakage), we also computed a within-exam cross-validated solvability, which was comparable; both estimates remain specific to these examination formats.

Result. Cross-exam solvability = 34.4% (item-bootstrap 95% CI 31.7 to 38.1; chance 25%); per exam MIR 40%, EIR 33%, PIR 32%, FIR 33%. Coefficients were dominated by option length (len_z = 0.287; longer options more often correct), with weaker cues has_absolute = −0.509 (absolutes → wrong), is_allnone = 0.47 (all-of-the-above → right), and stem_overlap = −0.34 (stem-echo → wrong); several features fired on very few options. The classifier did not outperform the longest-option heuristic (34.6%; Table S19), consistent with a length-dominated item-writing artefact. To test whether models exploit it, we partitioned full-coverage model accuracy by shortcut-solvability; because shortcut-solvable items are also easier for humans, we report a human-facility-adjusted contrast (shortcut coefficient −0.1449, P=.11), providing no clear evidence of differential association after adjustment, rather than proof that surface cues are never used. Model accuracy was 93.2% on shortcut-solvable versus 91.7% on shortcut-resistant items (Δ=1.6 pp, overlapping CIs); humans dropped 66% to 61%. These features are Spanish- and format-specific.

## M6. Pillar 5: Rationale stylometry: feature details and classifier

To characterize model explanatory prose we assembled 12,043 AI rationales (5343 Claude, 4974 GPT, 898 Gemini, 828 chatbot) alongside the 839 human stems, computing:

- burstiness (sentence-length variability);
- lexical diversity (type-token ratio, hapax ratio);
- sentence-length statistics (mean and variability);
- format/templating markers (newline rate, colon rate, list/heading markers);
- word count and output length;
- a corpus-internal perplexity index from a held-out 5-fold trigram model (a relative predictability index).

A random-forest classifier predicted the generating family from style alone, evaluated by 5-fold cross-validation on the de-duplicated subset of families with ≥100 samples (n=8183; majority-class baseline 56.1%).

Result. Family identifiable from style alone at 82.8% versus the majority-class baseline 56.1% (top features: word count, type-token ratio, newline rate, hapax ratio, colon rate, perplexity). Descriptive contrasts: AI burstiness −0.53 versus human stems −0.25; perplexity 89.8 (AI) versus 190.9 (human stems); templating absent in human stems. An exploratory human-versus-AI separability reached AUROC 0.96 but is genre-confounded.

Caveats. Output length and formatting are the dominant, generation-configuration-driven signals; the perplexity feature is partly circular (the n-gram model is trained on a Claude/GPT-dominated corpus); and Gemini carries a content confound (predominantly MIR, no PIR). The funder chatbot enters here only as a separate, non-ranked descriptive family. This pillar is explicitly not a claim that any official exam item is AI-written.

## M7. Single-run limitation and feasibility of a run-to-run probe

Each configuration was evaluated once, without repeated sampling or temperature variation; run-to-run stochastic variance is unquantified. Every psychometric estimate (KR-20, point-biserial discrimination, machine facility, the 80.7% ceiling, ρ=0.40, and all bootstrap CIs) rests on one stochastic draw per configuration; the psychometric bootstrap resamples items and cannot capture run-to-run variance. The error-concordance contrast instead resamples error events (Section M4), and audit intervals are conditional on the available judged records. These intervals do not represent total uncertainty.

A seed-and-temperature re-querying of GPT-5.2 and Gemini to estimate run-to-run variance was not feasible in-environment: the OpenAI and Google application programming interface (API) credentials required to re-query those models were not available to the secondary analysis (the original item-level responses were collected by the study team). We report two feasible proxies in their place:

1. Prompt-perturbation stability proxy. The panel difficulty-transfer ρ is stable across prompting frames (0.3961 neutral-only panel versus 0.3693 expert-only panel), and the mean absolute per-model accuracy shift between frames is 0.4 pp (max 0.9 pp), indicating small configuration instability under this perturbation.
2. Cross-judge agreement. The Opus-versus-Sonnet second-judge agreement (Section M8) assesses agreement for the audit labels; it does not estimate variability in the primary models' answers.

These are proxies, not substitutes, for a true run-to-run variance estimate. Reliability figures are reported to two significant figures, within-noise contrasts are not interpreted (reasoning-effort null, expert-versus-neutral differences, per-exam ρ heterogeneity 0.30 to 0.49), and every comparative claim is capped at the cohort-descriptive level.

## M8. Pillar 4: Multi-judge audit reliability regimes (full detail)

Two reliability regimes are kept distinct and never conflated.

- Triple-judge Fleiss κ. Computed on an n=59, MIR/EIR-only subset using three independent Opus judge instances: κ=0.7589 (2-of-3 agreement 0.9661).
- Two-judge Opus-versus-Sonnet Cohen κ on available paired records: failure-mode κ=0.4283 (agreement 0.5844, n=231 errors); clinically-dangerous κ=0.3765 (n=231 errors); reasoning-mode κ=0.6501 (n=236 correct rationales); and faithfulness κ=0.4181 (n=236 correct rationales). These paired records span all four examinations.

The second judge returned 231 usable error labels and 239 correct-rationale labels; 236 correct rationales matched the primary-judge sample for agreement analysis. Sonnet labeled 46.8% of errors as knowledge gaps, 90.8% of its correct-rationale pool as faithful (217/239), and 56.5% as factual recall (135/239). Danger flags were judge-sensitive: Opus flagged 16/253 errors (6.3%; bootstrap 95% CI 3.6 to 9.5), while Sonnet flagged 27/231 (11.7%). These percentages use different available error denominators and are conditional label rates, not estimates or bounds of patient risk. Only the triple-judge subset was restricted to MIR and EIR; the two-judge comparisons included PIR and FIR. The Claude-family judges may share systematic biases. A cross-vendor judge and clinician adjudication of flagged errors with a control sample are needed before any clinical-safety inference. Within-judge intervals omit uncertainty from judge selection, label validity and missing audit records.

## M9. Coverage matrix (Table S1)

System × exam × pillar coverage (response-observation counts) showing which systems entered the 12-configuration headline panel, which are descriptive-only (including the MIR-only Claude Opus 4.6 configuration and the funder chatbot), and where Gemini and the funder chatbot are excluded from each pillar. The 12-configuration headline panel comprises six model/effort settings (GPT-5.2 at low/medium/high reasoning effort, Claude Opus 4.5, Claude Sonnet 4.5, Claude Haiku 4.5) each under both a neutral and an expert prompt frame (6 × 2 = 12), each run once. The funder (Healthcademia) chatbot is excluded from the panel, from all comparative ranking, and from construct pillars 1 to 4; it appears only as a separate, non-ranked descriptive family in pillar 5, with its accuracy reported separately for transparency (pooled 80.4%; per-exam EIR 85.9%, FIR 75.4%, MIR 86.6%, PIR 74.0%).

## M10. Analyses added at first revision, in response to peer review

All analyses in this section were requested by the reviewers of the first submission and were computed from the same item-level results files as the main analyses. No new model runs were performed.

### M10.1 Sensitivity of the human-machine coupling to panel composition

The primary machine-facility panel comprises 12 configurations (6 model/effort settings x 2 prompt frames). Because those configurations are not independent, a reviewer asked whether the central correlation and the reported ceiling depend on that composition. We recomputed both under seven panel definitions, including one neutral condition per distinct model, vendor-balanced panels, and leave-one-model-family-out panels (Table S14). Spearman rho ranges from 0.31 to 0.40 and the AI ceiling from 80.7% to 92.9%. The modest correlations persist across these panel definitions. Their ceilings also change with panel size, and the 0.63 null expectation was calibrated for the primary 12-configuration panel; it is not a recalibrated null for each alternative panel.

### M10.2 Sensitivity of the disattenuation correction to the assumed reliabilities

The disattenuation correction requires reliabilities for both facility measures. The machine-panel KR-20 is not a conventional test reliability and the human-facility reliability is assumed rather than estimated, so we report the corrected estimate across a grid of plausible values rather than at a single point (Table S15). Across human reliabilities 0.90 to 0.98 and machine reliabilities 0.80 to 0.95, the disattenuated Pearson correlation ranges 0.440 to 0.500 and the disattenuated shared variance ranges 19.3% to 25.0%. The value reported in the main text, approximately 20%, is near the lower end of that assumed-reliability range.

### M10.3 Sensitivity to the handling of unparsed model responses

Eleven item-by-configuration observations returned no parsable option. All eleven are from the same configuration, Claude Opus 4.5 in the expert frame, and fall on 11 items (EIR 10, PIR 1); the machine-facility denominator therefore never falls below 11 of 12 for any item. Ten affected EIR items also lacked usable human facility and were already excluded from the 829 items with human facility. We compared excluding unparsed responses from the machine denominator and scoring them as incorrect on these 829 items; complete-case analysis additionally excluded the one affected PIR item, leaving 828 items. The resulting correlations are 0.398, 0.398 and 0.397 respectively, and the ceiling is 80.6%, 80.6% and 80.7%. The choice of rule does not affect any conclusion.

### M10.4 Examination-stratified and meta-analytic pooling

Because pooling items across four examinations could in principle create an aggregation artefact, we report the four within-examination correlations with confidence intervals and a fixed-effect meta-analytic summary on the Fisher z scale (Table S16). The pooled estimate is rho 0.39 (95% CI 0.33 to 0.45), with Q = 5.10 and I2 = 41%, against a naive pooled estimate of 0.40. The two agree to within 0.01, providing no indication that pooling alone accounts for the modest association in this cohort.

### M10.5 Trap items and hard-for-human, perfect-for-AI items are related but distinct sets

The trap-item count (147) numerically equals the count of hard-for-human, perfect-for-AI items (147). A reviewer asked whether the two metrics do separable analytical work. The cross-tabulation (Table S17) shows an intersection of 77 items, 70 items that are trap-only, 70 that are hard-and-perfect-only, and 611 that are neither, with phi = 0.42 (chi-square P < .001). The trap-only stratum illustrates the distinction: those 70 items have the lowest human facility of any stratum (0.21) yet AI panel facility falls to 0.57, whereas the hard-and-perfect stratum sits at 1.00 by construction. The two indicators therefore identify overlapping but different sets of items.

### M10.6 Image effect stratified by whether the image was supplied

MIR image items were administered to the multimodal pipeline with the image present, whereas EIR and FIR image-associated items were processed through the text-only pipeline without the image. Because image availability, examination and administration route are confounded in the pooled estimate, we refitted the marginal logistic model separately within each stratum (Table S18). Where the image was supplied, accuracy on image items was 89.9% versus 96.6% on non-image items, marginal odds ratio 0.33 (95% CI 0.21 to 0.53). Where the image was withheld, accuracy was 71.2% versus 95.1%, odds ratio 0.11 (95% CI 0.08 to 0.16). The pooled estimate combines different examinations and administration conditions. These associations cannot isolate visual-reasoning ability from missing information, examination content or administration route. A properly controlled estimate would require re-administering the EIR and FIR image items through a multimodal pipeline, which we identify as a committed next step.

### M10.7 Surface-feature classifier: cross-validation scheme and heuristic benchmark

Cross-validation was leave-one-exam-out. This is stricter than grouping folds by item, because every answer option of a given item lies wholly within a single examination, so no item and no option can straddle the training and validation partitions. We also benchmarked the classifier against the single strongest heuristic it could be exploiting (Table S19). Classifier item-level accuracy is 34.4% (289/839, 95% CI 31.3 to 37.7) against a 25% chance baseline, while simply selecting the longest option achieves 34.6% (290/839, 95% CI 31.4 to 37.8) and selecting position 1 achieves 22.5% (189/839). The classifier did not outperform the longest-option heuristic, and the finding is properly described as a length-dominated item-writing artefact rather than a broad surface-leakage construct. One of the eleven features, stem lexical overlap, is computed from the stem, so what is withheld from the classifier is the stem's medical meaning rather than every signal derived from the stem.

Table S14 (supplementary). Sensitivity of the human-machine difficulty coupling and of the AI ceiling to the composition of the machine-facility panel. Items are those with full coverage under the panel concerned. The final column counts items with at least one error (machine facility <1); the 150-item mixed-response sensitivity subset in Section M3 additionally excludes 10 all-incorrect items.

| **Panel definition** | **Configurations** | **n items** | **Spearman rho** | **AI ceiling** | **Items with ≥1 error** |
| --- | --- | --- | --- | --- | --- |
| Primary: 6 model/effort settings x 2 frames | 12 | 828 | 0.397 | 80.7% | 160 |
| Neutral frame only | 6 | 829 | 0.396 | 82.1% | 148 |
| Expert frame only | 6 | 828 | 0.369 | 83.2% | 139 |
| Claude family only | 6 | 828 | 0.393 | 81.3% | 155 |
| GPT family only | 6 | 829 | 0.309 | 92.0% | 66 |
| Vendor-balanced: 3 Claude and 1 GPT, neutral | 4 | 829 | 0.398 | 82.6% | 144 |
| Vendor-balanced: best configuration per vendor, neutral | 2 | 829 | 0.315 | 92.9% | 59 |

Table S15 (supplementary). Disattenuated Pearson correlation and disattenuated shared variance across plausible reliability assumptions for the human and machine facility measures. Observed Pearson r = 0.424.

| **Assumed human reliability** | **Assumed machine reliability** | **Disattenuated r** | **Disattenuated shared variance** |
| --- | --- | --- | --- |
| 0.90 | 0.80 | 0.500 | 25.0% |
| 0.90 | 0.86 | 0.482 | 23.3% |
| 0.90 | 0.93 | 0.464 | 21.5% |
| 0.90 | 0.95 | 0.459 | 21.1% |
| 0.95 | 0.80 | 0.487 | 23.7% |
| 0.95 | 0.86 | 0.469 | 22.0% |
| 0.95 | 0.93 | 0.451 | 20.4% |
| 0.95 | 0.95 | 0.447 | 19.9% |
| 0.98 | 0.80 | 0.479 | 23.0% |
| 0.98 | 0.86 | 0.462 | 21.4% |
| 0.98 | 0.93 | 0.444 | 19.8% |
| 0.98 | 0.95 | 0.440 | 19.3% |

Table S16 (supplementary). Within-examination correlations between human and machine item facility, and fixed-effect meta-analytic pooling on the Fisher z scale. Examinations are listed in the fixed order used throughout.

| **Examination** | **n items** | **Spearman rho** | **95% CI** |
| --- | --- | --- | --- |
| MIR | 203 | 0.301 | 0.171 to 0.422 |
| EIR | 210 | 0.380 | 0.258 to 0.490 |
| PIR | 209 | 0.487 | 0.377 to 0.585 |
| FIR | 206 | 0.382 | 0.258 to 0.493 |
| Fixed-effect pooled | 828 | 0.390 | 0.331 to 0.447 |
| Naive pooled, ignoring examination | 828 | 0.397 | 0.336 to 0.453 |

Heterogeneity across examinations: Q = 5.10 on 3 degrees of freedom, I2 = 41%.

Table S17 (supplementary). Cross-tabulation of trap items against hard-for-human, perfect-for-AI items, with mean human and machine facility in each cell. Trap items are those on which the human plurality selected a wrong option. Hard-for-human, perfect-for-AI items are those with human facility below 0.50 answered correctly by all 12 configurations.

| **Stratum** | **n items** | **Mean human facility** | **Mean AI panel facility** |
| --- | --- | --- | --- |
| Trap and hard-for-human, perfect-for-AI | 77 | 0.278 | 1.000 |
| Trap only | 70 | 0.206 | 0.570 |
| Hard-for-human, perfect-for-AI only | 70 | 0.426 | 1.000 |
| Neither | 611 | 0.740 | 0.958 |

Association between the two indicators: phi = 0.42, chi-square P < .001. Union of the two sets 217 items, intersection 77 items.

Table S18 (supplementary). Image-associated item effect, stratified by whether the image was supplied to the model. Marginal (population-averaged) logistic models adjusting for human facility.

| **Stratum** | **Administration** | **Accuracy on image items** | **Accuracy on non-image items** | **Marginal odds ratio** | **95% CI** |
| --- | --- | --- | --- | --- | --- |
| MIR | Image supplied to the multimodal pipeline | 89.9% | 96.6% | 0.33 | 0.21 to 0.53 |
| EIR and FIR | Image withheld, text-only pipeline | 71.2% | 95.1% | 0.11 | 0.08 to 0.16 |
| Pooled across all four examinations | Mixed | 80.6% | 94.0% | 0.21 | 0.16 to 0.26 |

PIR contains no image-associated items. Image-associated observations: 288 in each supplied/withheld stratum. The pooled model includes all four examinations (9947 response observations): 464/576 image-associated responses were correct (80.6%) versus 8811/9371 non-image responses (94.0%).

Table S19 (supplementary). Surface-feature classifier benchmarked against single-heuristic baselines, by examination. Item-level accuracy in recovering the keyed option.

| **Examination** | **n items** | **Classifier** | **Longest option** | **Position 1** | **Chance** |
| --- | --- | --- | --- | --- | --- |
| MIR | 203 | 39.9% | 39.4% | 20.2% | 25.0% |
| EIR | 220 | 32.7% | 32.7% | 22.3% | 25.0% |
| PIR | 210 | 32.4% | 31.9% | 26.7% | 25.0% |
| FIR | 206 | 33.0% | 34.5% | 20.9% | 25.0% |
| All | 839 | 34.4% (289/839) | 34.6% (290/839) | 22.5% (189/839) | 25.0% |

Classifier 95% CI 31.3 to 37.7; longest-option 95% CI 31.4 to 37.8 (Wilson).

## Supplementary references

1. Geirhos R, Jacobsen J-H, Michaelis C, Zemel R, Brendel W, Bethge M, Wichmann FA. Shortcut learning in deep neural networks. Nat Mach Intell. 2020;2(11):665-673. doi:10.1038/s42256-020-00257-z
2. Lapuschkin S, Wäldchen S, Binder A, Montavon G, Samek W, Müller K-R. Unmasking Clever Hans predictors and assessing what machines really learn. Nat Commun. 2019;10:1096. doi:10.1038/s41467-019-08987-4
3. Balepur N, Ravichander A, Rudinger R. Artifacts or abduction: how do LLMs answer multiple-choice questions without the question? In: Proc 62nd Annu Meet Assoc Comput Linguist (ACL). 2024. arXiv:2402.12483.
4. Messick S. Validity of psychological assessment: validation of inferences from persons' responses and performances as scientific inquiry into score meaning. Am Psychol. 1995;50(9):741-749. doi:10.1037/0003-066X.50.9.741
5. Kane MT. Validating the interpretations and uses of test scores. J Educ Meas. 2013;50(1):1-73. doi:10.1111/jedm.12000
6. Lord FM, Novick MR. Statistical Theories of Mental Test Scores. Reading, MA: Addison-Wesley; 1968.
7. Embretson SE, Reise SP. Item Response Theory for Psychologists. Mahwah, NJ: Lawrence Erlbaum Associates; 2000.

# **Part S2. Supplementary Tables and Coverage Matrix**

Table S1 (supplementary). Coverage matrix for the listed systems: response observations across available prompt frames, headline-panel membership, and pillars entered. Counts are responses, not unique items; Table S2 provides model-by-frame accuracy details. The funder chatbot is excluded from the headline panel and pillars 1 to 4 and appears only as a separate descriptive family in pillar 5.

| **System** | **Headline panel** | **MIR** | **EIR** | **PIR** | **FIR** | **Pillars entered** |
| --- | --- | --- | --- | --- | --- | --- |
| GPT-5.2 high | Yes | 406 | 440 | 420 | 412 | 1 to 5 |
| GPT-5.2 medium | Yes | 406 | 440 | 420 | 412 | 1 to 3, 5 |
| GPT-5.2 low | Yes | 406 | 440 | 420 | 412 | 1 to 3, 5 |
| Claude Opus 4.5 | Yes | 406 | 430 | 419 | 412 | 1 to 5 |
| Claude Sonnet 4.5 | Yes | 406 | 440 | 420 | 412 | 1 to 5 |
| Claude Haiku 4.5 | Yes | 406 | 440 | 420 | 412 | 1 to 5 |
| Gemini 3 Flash | No (MIR-focused) | 406 | 30 | — | 28 | descriptive |
| Healthcademia chatbot | No (funder; excluded) | 201 | 198 | 204 | 199 | 5 (descriptive) |
| OpenEvidence | No (MIR only) | 172 | — | — | — | descriptive |

## Table S2. Accuracy by model configuration and examination (including partial coverage)

| **family** | **model** | **prompt** | **exam** | **n** | **correct** | **accuracy_pct** |
| --- | --- | --- | --- | --- | --- | --- |
| Claude | Claude Haiku 4.5 | expert | EIR | 220 | 184 | 83.6 |
| Claude | Claude Haiku 4.5 | neutral | EIR | 220 | 188 | 85.5 |
| Claude | Claude Opus 4.5 | expert | EIR | 210 | 200 | 95.2 |
| Claude | Claude Opus 4.5 | neutral | EIR | 220 | 198 | 90.0 |
| Claude | Claude Sonnet 4.5 | expert | EIR | 220 | 194 | 88.2 |
| Claude | Claude Sonnet 4.5 | neutral | EIR | 220 | 189 | 85.9 |
| GPT | GPT-5.2 high | expert | EIR | 220 | 197 | 89.5 |
| GPT | GPT-5.2 high | neutral | EIR | 220 | 199 | 90.5 |
| GPT | GPT-5.2 low | expert | EIR | 220 | 196 | 89.1 |
| GPT | GPT-5.2 low | neutral | EIR | 220 | 200 | 90.9 |
| GPT | GPT-5.2 medium | expert | EIR | 220 | 199 | 90.5 |
| GPT | GPT-5.2 medium | neutral | EIR | 220 | 196 | 89.1 |
| Gemini | Gemini 3 Flash | expert | EIR | 20 | 8 | 40.0 |
| Gemini | Gemini 3 Flash | neutral | EIR | 10 | 8 | 80.0 |
| Claude | Claude Haiku 4.5 | expert | FIR | 206 | 182 | 88.3 |
| Claude | Claude Haiku 4.5 | neutral | FIR | 206 | 173 | 84.0 |
| Claude | Claude Opus 4.5 | expert | FIR | 206 | 197 | 95.6 |
| Claude | Claude Opus 4.5 | neutral | FIR | 206 | 198 | 96.1 |
| Claude | Claude Sonnet 4.5 | expert | FIR | 206 | 197 | 95.6 |
| Claude | Claude Sonnet 4.5 | neutral | FIR | 206 | 195 | 94.7 |
| GPT | GPT-5.2 high | expert | FIR | 206 | 202 | 98.1 |
| GPT | GPT-5.2 high | neutral | FIR | 206 | 200 | 97.1 |
| GPT | GPT-5.2 low | expert | FIR | 206 | 197 | 95.6 |
| GPT | GPT-5.2 low | neutral | FIR | 206 | 197 | 95.6 |
| GPT | GPT-5.2 medium | expert | FIR | 206 | 201 | 97.6 |
| GPT | GPT-5.2 medium | neutral | FIR | 206 | 199 | 96.6 |
| Gemini | Gemini 3 Flash | expert | FIR | 14 | 14 | 100.0 |
| Gemini | Gemini 3 Flash | neutral | FIR | 14 | 13 | 92.9 |
| Claude | Claude Haiku 4.5 | expert | MIR | 203 | 178 | 87.7 |
| Claude | Claude Haiku 4.5 | neutral | MIR | 203 | 181 | 89.2 |
| Claude | Claude Opus 4.5 | expert | MIR | 203 | 195 | 96.1 |
| Claude | Claude Opus 4.5 | neutral | MIR | 203 | 198 | 97.5 |
| Claude | Claude Opus 4.6 | expert | MIR | 203 | 196 | 96.6 |
| Claude | Claude Opus 4.6 | neutral | MIR | 203 | 195 | 96.1 |
| Claude | Claude Sonnet 4.5 | expert | MIR | 203 | 193 | 95.1 |
| Claude | Claude Sonnet 4.5 | neutral | MIR | 203 | 197 | 97.0 |
| GPT | GPT-5.2 high | expert | MIR | 203 | 200 | 98.5 |
| GPT | GPT-5.2 high | neutral | MIR | 203 | 202 | 99.5 |
| GPT | GPT-5.2 low | expert | MIR | 203 | 199 | 98.0 |
| GPT | GPT-5.2 low | neutral | MIR | 203 | 196 | 96.6 |
| GPT | GPT-5.2 medium | expert | MIR | 203 | 197 | 97.0 |
| GPT | GPT-5.2 medium | neutral | MIR | 203 | 199 | 98.0 |
| Gemini | Gemini 3 Flash | expert | MIR | 203 | 201 | 99.0 |
| Gemini | Gemini 3 Flash | neutral | MIR | 203 | 199 | 98.0 |
| Claude | Claude Haiku 4.5 | expert | PIR | 210 | 175 | 83.3 |
| Claude | Claude Haiku 4.5 | neutral | PIR | 210 | 173 | 82.4 |
| Claude | Claude Opus 4.5 | expert | PIR | 209 | 189 | 90.4 |
| Claude | Claude Opus 4.5 | neutral | PIR | 210 | 190 | 90.5 |
| Claude | Claude Sonnet 4.5 | expert | PIR | 210 | 186 | 88.6 |
| Claude | Claude Sonnet 4.5 | neutral | PIR | 210 | 186 | 88.6 |
| GPT | GPT-5.2 high | expert | PIR | 210 | 192 | 91.4 |
| GPT | GPT-5.2 high | neutral | PIR | 210 | 194 | 92.4 |
| GPT | GPT-5.2 low | expert | PIR | 210 | 195 | 92.9 |
| GPT | GPT-5.2 low | neutral | PIR | 210 | 194 | 92.4 |
| GPT | GPT-5.2 medium | expert | PIR | 210 | 193 | 91.9 |
| GPT | GPT-5.2 medium | neutral | PIR | 210 | 195 | 92.9 |

## Table S3. Confidence calibration by model configuration

| **Configuration** | **Family** | **n** | **Acc.** | **Mean conf. correct (%)** | **Mean conf. incorrect (%)** | **Wrong** | **Gap** | **AUC** | **ECE** | **Stable** | **Overconf** |
| --- | --- | --- | --- | --- | --- | --- | --- | --- | --- | --- | --- |
| GPT-5.2 high (expert) | GPT | 831 | 0.942 | 90.15 | 62.52 | 48 | 27.63 | 0.9 | 0.058 | True | -5.67 |
| GPT-5.2 medium (expert) | GPT | 831 | 0.941 | 90.37 | 59.92 | 49 | 30.46 | 0.891 | 0.06 | True | -5.52 |
| GPT-5.2 high (neutral) | GPT | 831 | 0.947 | 89.92 | 61.34 | 44 | 28.58 | 0.887 | 0.065 | True | -6.3 |
| GPT-5.2 low (expert) | GPT | 831 | 0.939 | 90.17 | 62.22 | 51 | 27.96 | 0.873 | 0.058 | True | -5.4 |
| GPT-5.2 low (neutral) | GPT | 831 | 0.939 | 90.14 | 61.29 | 51 | 28.84 | 0.871 | 0.055 | True | -5.5 |
| GPT-5.2 medium (neutral) | GPT | 831 | 0.94 | 89.95 | 64.22 | 50 | 25.73 | 0.871 | 0.056 | True | -5.58 |
| Gemini 3 Flash (expert) | Gemini | 219 | 0.986 | 91.49 | 93.33 | 3 | -1.85 | 0.845 | 0.071 | False | -7.12 |
| Claude Opus 4.5 (neutral) | Claude | 828 | 0.937 | 88.04 | 69.21 | 52 | 18.83 | 0.84 | 0.075 | True | -6.86 |
| Claude Sonnet 4.5 (neutral) | Claude | 831 | 0.915 | 92.01 | 73.56 | 71 | 18.45 | 0.831 | 0.049 | True | -1.02 |
| Claude Opus 4.5 (expert) | Claude | 820 | 0.944 | 88.38 | 77.04 | 46 | 11.34 | 0.818 | 0.07 | True | -6.64 |
| Claude Sonnet 4.5 (expert) | Claude | 831 | 0.918 | 92.96 | 73.49 | 68 | 19.48 | 0.796 | 0.03 | True | -0.45 |
| Gemini 3 Flash (neutral) | Gemini | 219 | 0.973 | 91.17 | 95.0 | 6 | -3.83 | 0.786 | 0.084 | False | -5.99 |
| Claude Haiku 4.5 (neutral) | Claude | 831 | 0.853 | 88.65 | 73.87 | 122 | 14.78 | 0.761 | 0.046 | True | 1.16 |
| Claude Haiku 4.5 (expert) | Claude | 831 | 0.859 | 89.23 | 74.18 | 117 | 15.05 | 0.756 | 0.04 | True | 1.19 |

## Table S4. Response latency by model configuration

| **display** | **n** | **median_ms** | **mean_ms** | **acc** |
| --- | --- | --- | --- | --- |
| Claude Haiku 4.5 (neutral) | 781 | 1562.0 | 1687.2 | 0.886 |
| Claude Haiku 4.5 (expert) | 781 | 1612.0 | 1743.9 | 0.887 |
| GPT-5.2 low (neutral) | 781 | 2757.0 | 3175.2 | 0.958 |
| GPT-5.2 low (expert) | 781 | 2882.0 | 3294.0 | 0.958 |
| Claude Sonnet 4.5 (neutral) | 781 | 3079.0 | 3152.7 | 0.94 |
| Gemini 3 Flash (neutral) | 179 | 3129.0 | 3525.8 | 0.983 |
| GPT-5.2 medium (neutral) | 781 | 3145.0 | 3756.5 | 0.955 |
| Claude Sonnet 4.5 (expert) | 781 | 3157.0 | 3216.5 | 0.937 |
| Gemini 3 Flash (expert) | 179 | 3170.0 | 3657.8 | 0.994 |
| GPT-5.2 high (neutral) | 781 | 3388.0 | 4119.6 | 0.96 |
| GPT-5.2 medium (expert) | 781 | 3396.0 | 4053.4 | 0.955 |
| Claude Opus 4.5 (neutral) | 781 | 3455.0 | 3521.9 | 0.946 |
| Claude Opus 4.5 (expert) | 780 | 3552.0 | 3618.7 | 0.946 |
| GPT-5.2 high (expert) | 781 | 3745.0 | 4717.6 | 0.954 |

## Table S5. Per-model stylometric signatures

| **Model** | **Burstiness** | **Perplexity** | **TTR** | **Mean sentence length** | **Numbered list** | **Bold per 1000** | **Words** |
| --- | --- | --- | --- | --- | --- | --- | --- |
| Claude Haiku 4.5 | -0.598 | 123.415 | 0.798 | 25.542 | 0.024 | 0.192 | 57.127 |
| Claude Opus 4.5 | -0.591 | 61.358 | 0.785 | 27.181 | 0.013 | 0.12 | 60.657 |
| Claude Opus 4.6 | -0.294 | 490.439 | 0.685 | 14.02 | 0.142 | 3.964 | 308.708 |
| Claude Sonnet 4.5 | -0.582 | 71.125 | 0.815 | 25.444 | 0.012 | 0.026 | 48.948 |
| GPT-5.2 high | -0.575 | 62.999 | 0.864 | 23.541 | 0.013 | 0.062 | 37.174 |
| GPT-5.2 low | -0.578 | 55.643 | 0.864 | 22.47 | 0.008 | 0.026 | 35.6 |
| GPT-5.2 medium | -0.566 | 60.852 | 0.864 | 22.956 | 0.014 | 0.081 | 37.872 |
| Gemini 3 Flash | -0.481 | 86.522 | 0.829 | 27.95 | 0.053 | 0.002 | 54.954 |
| Healthcademia chatbot | -0.279 | 109.318 | 0.642 | 20.607 | 0.021 | 0.0 | 115.155 |

## Table S6. Subject-domain accuracy vs human candidate facility

| **exam** | **asignatura** | **n_items** | **llm_acc** | **candidate_facility** |
| --- | --- | --- | --- | --- |
| EIR | SP | 10 | 71.7 | 45.1 |
| EIR | CD | 8 | 79.2 | 45.8 |
| EIR | EC | 15 | 81.1 | 47.6 |
| EIR | GR | 6 | 83.3 | 42.2 |
| EIR | UR | 2 | 83.3 | 29.6 |
| EIR | HF | 15 | 84.4 | 43.9 |
| EIR | NM | 6 | 86.1 | 58.9 |
| EIR | PT | 5 | 86.7 | 62.6 |
| EIR | EL | 14 | 92.3 | 70.6 |
| EIR | PS | 6 | 94.4 | 47.8 |
| EIR | ET | 8 | 95.8 | 50.5 |
| EIR | OR | 2 | 95.8 | 25.3 |
| EIR | UE | 11 | 96.2 | 78.4 |
| EIR | NR | 4 | 97.9 | 50.5 |
| EIR | ND | 11 | 99.2 | 46.6 |
| EIR | NF | 10 | 99.2 | 54.4 |
| EIR | AS | 7 | 100.0 | 64.9 |
| EIR | BQ | 10 | 100.0 | 73.8 |
| EIR | DG | 4 | 100.0 | 47.5 |
| EIR | DM | 1 | 100.0 | 75.3 |
| EIR | ED | 5 | 100.0 | 61.6 |
| EIR | EM | 9 | 100.0 | 68.9 |
| EIR | EP | 10 | 100.0 | 55.6 |
| EIR | FC | 9 | 100.0 | 54.9 |
| EIR | HT | 1 | 100.0 | 13.1 |
| EIR | ON | 4 | 100.0 | 43.7 |
| EIR | SM | 15 | 100.0 | 64.9 |
| EIR | TR | 2 | 100.0 | 62.6 |
| FIR | QF | 18 | 81.9 | 61.4 |
| FIR | FG | 3 | 88.9 | 33.5 |
| FIR | BM | 11 | 90.2 | 60.9 |
| FIR | FS | 34 | 92.9 | 63.6 |
| FIR | TF | 10 | 93.3 | 56.8 |
| FIR | TI | 15 | 93.3 | 55.0 |
| FIR | LG | 6 | 95.8 | 59.0 |
| FIR | MC | 25 | 96.0 | 61.7 |
| FIR | BF | 13 | 96.2 | 70.2 |
| FIR | QM | 9 | 96.3 | 57.5 |
| FIR | BQ | 13 | 98.7 | 53.8 |
| FIR | FC | 26 | 99.4 | 69.1 |
| FIR | BR | 2 | 100.0 | 48.1 |
| FIR | ET | 5 | 100.0 | 55.0 |
| FIR | IM | 8 | 100.0 | 57.9 |
| FIR | SP | 3 | 100.0 | 67.4 |
| FIR | TX | 5 | 100.0 | 66.5 |
| MIR | HM | 6 | 88.9 | 65.2 |
| MIR | NM | 12 | 89.6 | 70.9 |
| MIR | ORL | 6 | 90.3 | 66.4 |
| MIR | TM | 9 | 92.6 | 64.6 |
| MIR | DM | 7 | 92.9 | 53.1 |
| MIR | DG | 24 | 94.8 | 69.3 |
| MIR | MC | 26 | 95.5 | 61.8 |
| MIR | CD | 20 | 95.8 | 66.2 |
| MIR | NF | 9 | 96.3 | 68.4 |
| MIR | IF | 10 | 96.7 | 52.4 |
| MIR | PD | 13 | 97.4 | 76.4 |
| MIR | ED | 8 | 97.9 | 60.6 |
| MIR | RM | 8 | 97.9 | 65.0 |
| MIR | GC | 9 | 98.1 | 62.5 |
| MIR | ET | 2 | 100.0 | 53.8 |
| MIR | IM | 3 | 100.0 | 74.3 |
| MIR | NR | 13 | 100.0 | 64.2 |
| MIR | OF | 4 | 100.0 | 40.8 |
| MIR | PSQ | 8 | 100.0 | 72.8 |
| MIR | UR | 6 | 100.0 | 76.2 |

## Table S7. Two difficulty axes & AI-panel reliability, by exam

| **exam** | **spearman_human_vs_machine** | **n** | **ai_panel_KR20** |
| --- | --- | --- | --- |
| MIR | 0.301 | 203 | 0.863 |
| EIR | 0.38 | 210 | 0.953 |
| PIR | 0.488 | 209 | 0.932 |
| FIR | 0.382 | 206 | 0.876 |

## Table S8. Trap items (human plurality wrong), by exam

| **exam** | **n_trap_items** | **ai_facility_trap** | **ai_facility_nontrap** | **human_facility_trap** |
| --- | --- | --- | --- | --- |
| MIR | 22 | 0.852 | 0.972 | 0.243 |
| EIR | 35 | 0.802 | 0.954 | 0.239 |
| PIR | 54 | 0.733 | 0.955 | 0.241 |
| FIR | 36 | 0.847 | 0.967 | 0.253 |
| ALL | 147 | 0.795 | 0.962 | 0.244 |

## Table S9. Construct-irrelevant surface solvability, by exam

| **exam** | **shortcut_acc** | **longest_answer_acc** |
| --- | --- | --- |
| MIR | 0.399 | 0.394 |
| EIR | 0.327 | 0.327 |
| PIR | 0.324 | 0.319 |
| FIR | 0.33 | 0.345 |

## Table S10. Surface-feature classifier coefficients

| **feature** | **coef** |
| --- | --- |
| has_absolute | -0.509 |
| is_allnone | 0.47 |
| stem_overlap | -0.34 |
| len_z | 0.287 |
| has_negation | -0.174 |
| is_longest | 0.155 |
| has_number | -0.099 |
| is_shortest | 0.069 |
| position | -0.051 |
| word_len | 0.024 |
| char_len | -0.003 |

## Table S11. Audited failure-mode distribution, by exam

| **Exam** | **Knowledge gap** | **Reasoning error** | **Vignette misread** | **Fine distractor** | **Item flaw or keying** | **Visual dependent** | **Hallucination** |
| --- | --- | --- | --- | --- | --- | --- | --- |
| MIR | 0.438 | 0.281 | 0.125 | 0.062 | 0.062 | 0.031 | 0.0 |
| EIR | 0.333 | 0.227 | 0.03 | 0.121 | 0.091 | 0.182 | 0.015 |
| PIR | 0.423 | 0.258 | 0.072 | 0.237 | 0.0 | 0.0 | 0.01 |
| FIR | 0.379 | 0.328 | 0.0 | 0.052 | 0.069 | 0.138 | 0.034 |

## Table S12. Audited failure-mode distribution, by model

| **Model** | **Knowledge gap** | **Reasoning error** | **Fine distractor** | **Visual dependent** | **Vignette misread** | **Hallucination** | **Item flaw or keying** |
| --- | --- | --- | --- | --- | --- | --- | --- |
| Claude Haiku 4.5 | 0.474 | 0.263 | 0.105 | 0.079 | 0.035 | 0.026 | 0.018 |
| Claude Opus 4.5 | 0.422 | 0.244 | 0.111 | 0.067 | 0.067 | 0.0 | 0.089 |
| Claude Sonnet 4.5 | 0.3 | 0.283 | 0.167 | 0.1 | 0.067 | 0.017 | 0.067 |
| GPT-5.2 high | 0.235 | 0.294 | 0.265 | 0.088 | 0.059 | 0.0 | 0.059 |

## Table S13. Rationale faithfulness, by exam

| **exam** | **faithful** | **partially_faithful** | **unfaithful_but_correct** | **unverifiable** |
| --- | --- | --- | --- | --- |
| MIR | 0.967 | 0.033 | 0.0 | 0.0 |
| EIR | 0.847 | 0.136 | 0.017 | 0.0 |
| PIR | 0.933 | 0.05 | 0.0 | 0.017 |
| FIR | 0.949 | 0.034 | 0.017 | 0.0 |

# **Part S3. LLM-Judge Rubric and Prompts (Generative-AI Prompts)**

This appendix documents the generative-AI prompts and rubric used in the multi-judge audit (Methods, Pillar 4), provided for transparency and reproducibility per JMIR policy on disclosing generative-AI prompts and responses. Judges were large language models (primary: Anthropic Claude Opus; second judge: Anthropic Claude Sonnet) applying a fixed, human-authored rubric, returning structured JSON against a fixed schema. No cross-vendor (non-Claude) judge was accessible in the analysis environment; this is a stated limitation and committed next step.

## A. Failure-mode rubric (eight mutually exclusive primary categories)

Each incorrectly answered item (the model selected an option other than the official key) was assigned exactly one primary failure mode:

- knowledge_gap: lacked a specific fact, drug, dose, guideline, or definition.
- reasoning_error: had the relevant facts but integrated/inferred them incorrectly.
- vignette_misread: ignored or misinterpreted a decisive datum stated in the stem (laboratory value, age, timing, keyword).
- fine_distractor: chose a clinically plausible near-miss requiring a subtle discrimination from the correct option.
- visual_dependent: the item hinges on an image the model could not properly use.
- item_flaw_or_keying: the item is genuinely ambiguous or the official key is disputable, so the model's answer is defensible.
- hallucination: invented or asserted a false fact/guideline to justify the answer.
- spanish_language: tripped on Spanish-specific phrasing, negation, or idiom.

A binary clinically_dangerous flag was set when acting on the model's answer instead of the official answer could plausibly harm a patient. Judges were instructed to judge independently and not to assume the official key was always correct.

## B. Failure-mode judge prompt (verbatim)

You are an expert physician-examiner auditing why a frontier AI model answered a Spanish health-professions specialty-access exam item INCORRECTLY (it chose model_answer; the official correct answer is official). For each item: read the model's rationale and choice and classify the single best primary failure mode from the rubric below; set clinically_dangerous=true if acting on model_answer instead of official could plausibly harm a patient; give judge_confidence (low/medium/high) and a one-sentence reason. Do not assume the official key is always right (use item_flaw_or_keying when warranted). Return one structured record per item (uid, model, primary_failure, secondary_flags, clinically_dangerous, judge_confidence, reason).

## C. Faithfulness rubric and judge prompt (correct answers)

For a stratified sample of correct answers (GPT-5.2-high; ~60 per exam), each rationale was classified as:

- faithfulness: faithful (reasoning correctly and sufficiently justifies the answer) | partially_faithful | unfaithful_but_correct (reasoning wrong/circular/irrelevant yet the answer is right) | unverifiable.
- reasoning_mode: clinical_reasoning | factual_recall | elimination | restates_answer | test_heuristic.
- contains_incorrect_statement: true if the rationale asserts any factually wrong statement even though the final answer is correct.

You are an expert physician-examiner auditing whether a frontier AI model's rationale for a CORRECT answer is sound (right-for-the-right-reasons) or merely arrives at the right option by weak/irrelevant reasoning. For each item classify faithfulness, dominant reasoning_mode, and contains_incorrect_statement, with a one-sentence reason. Return one structured record per item (uid, faithfulness, reasoning_mode, contains_incorrect_statement, reason).

## D. Reliability design

A triple-judged reliability subset (n=59; MIR and EIR items) was scored by three independent judge instances (Fleiss κ for the primary failure mode). The primary judge labeled 253 errors and 238 correct rationales. The second judge (Claude Sonnet) yielded 231 error records and 239 correct-rationale records. Paired cross-model agreement used 231 errors and 236 correct rationales spanning all four examinations (Cohen κ per construct; Section M8 and main Table 3). These are available-record analyses, not complete re-scoring of both primary samples.

## E. Two prompting frames used in the primary evaluation (not the audit)

The primary-panel configurations used two frames: a neutral frame (item stem + four options only) and an expert-role frame (a brief instruction to answer as a domain specialist). These frames define the 12-configuration machine-facility panel (6 model/effort settings × 2 frames).
