# Supplementary material for: Construct Validity of Frontier Large Language Models on 4 Spanish Health Professions Specialty-Access Examinations (MIR, EIR, PIR, and FIR): Cross-Sectional Benchmarking and Psychometric Study
Source: JMIR Med Educ. 2026 Oct 29;12:e106199. doi: 10.2196/106199 (PMC13718280; doi:10.2196/106199)
Supplement: Supplementary file 2 [file mededu-v12-e106199-s002.docx]

Multimedia Appendix 2

Correction script and reproduction instructions for the declared model panel

JMIR Medical Education manuscript 106199

This appendix provides the exact correction script for secondary analyses restricted to the declared panel of six model settings under neutral and expert prompts. It explains how to reproduce the corrected estimates from the public archived data and supporting source code. The script first verifies the archived unfiltered estimates, then applies the same estimators to the 12 declared configurations.

The corrected difference between observed top-distractor selection and the plausibility-weighted null is −1.25 percentage points (95% error-event bootstrap CI −4.53 to 2.19). The interval includes zero and supports neither systematic divergence nor equivalence. The primary item-facility correlation is unaffected by this correction.

# Scope and archived sources

The primary panel comprises Claude Opus 4.5, Claude Sonnet 4.5, Claude Haiku 4.5, and GPT-5.2 at low, medium, and high reasoning effort, each with neutral and expert prompts. The corrected panel contains 10,057 parsed responses. The replay requires no new model queries, examination text, image annexes, or additional clinical labels.

The original data and supporting analysis files are available in the public Zenodo deposit. The version-specific record used for this replay is:

https://doi.org/10.5281/zenodo.22100935

The concept DOI cited in the manuscript is https://doi.org/10.5281/zenodo.22100934. It resolves to this archived version. The deposit file is Zenodo_deposit_106199.zip (3,018,954 bytes; MD5 1d956520bfaae7fcfde65a3f9e8e6122). Public access and the seven required source-file hashes were verified on October 6, 2026.

The data and original supporting scripts remain in the repository; they are not embedded in this Word document. This appendix contains the complete correction script and the instructions needed to use those archived files.

# Reproduction instructions

Requirements: Python 3.11 or later with numpy, pandas, scipy, scikit-learn, and statsmodels. The replay was developed with Python 3.13. The source workbook is not needed because the workbook-loading functions are not called by this replay.

1. Download and extract Zenodo_deposit_106199.zip from the version-specific record above. Create a fresh working directory and arrange the following files. Copy the three scripts from code/ into analysis/ and the four data files from data/ into analysis/out/.

| **Source path in the Zenodo archive** | **Destination in the working directory** |
| --- | --- |
| code/common.py | analysis/common.py |
| code/d1_robust.py | analysis/d1_robust.py |
| code/d1_psychometrics.py | analysis/d1_psychometrics.py |
| data/long.csv | analysis/out/long.csv |
| data/item_master.csv | analysis/out/item_master.csv |
| data/results_core.json | analysis/out/results_core.json |
| data/results_d1.json | analysis/out/results_d1.json |

2. Create revision2/qa/ inside the same working directory. Copy the complete source listing at the end of this appendix into a UTF-8 plain-text file named correct_declared_panel.py in that directory. Preserve the indentation, straight quotation marks, and code. Wrapped display lines in Word are not additional source lines.

3. From the working directory, run:

python revision2/qa/correct_declared_panel.py

4. Successful execution writes revision2/qa/corrected_numbers.json and prints the corrected distractor, uncertainty, and calibration summaries. The assertions stop execution if the archived unfiltered estimates are not reproduced exactly. The source listing can also be extracted from the Word paragraphs styled Source code, excluding the one-line run command above.

# What the replay executes

The correction script imports the archived common.py module for its output-directory definition. It executes the prefix of d1_robust.py ending immediately before the trap-gap bootstrap section, first with all archived observations and then with the declared 12-configuration filter. This preserves the seed (20260621), preceding random draws, and individual-error resampling unit used by the original bootstrap.

The script separately reuses the error-concordance and entropy–confidence block from d1_psychometrics.py. It also restricts archived calibration and latency summaries to the declared panel and verifies image-associated odds ratios from logistic regression adjusted for human facility. The original source files and data are read without modification. The only generated file is corrected_numbers.json in the replay directory.

This is a targeted replay of the panel-selection corrections. It does not rerun every original analysis or regenerate the entire manuscript. The 2,000-replicate distractor bootstrap resamples individual errors and does not account for correlation among responses to the same item or related models, nor for variation across repeated model runs.

# Expected corrected estimates

| **Quantity** | **Corrected value** |
| --- | --- |
| Declared model configurations | 12 |
| Parsed model responses in the declared panel | 10,057 |
| Eligible incorrect model responses | 672 |
| Selection of the most popular human distractor | 428/672 (63.69%) |
| Plausibility-weighted expected selection | 64.94% |
| Observed minus expected selection | −1.25 percentage points |
| 95% error-event bootstrap confidence interval | −4.53 to 2.19 percentage points |
| Selection of the human plurality option among errors | 246/672 (36.61%) |
| Mean within-item wrong-option Pearson correlation | 0.4998 across 161 items |
| Human entropy versus model confidence | Spearman ρ=−0.3049; n=9851 |
| Underconfident configurations | 10/12 |
| Expected calibration error range | 0.0299 to 0.0753 |

The 64.94% null expectation is the mean of event-specific probabilities and has no integer n/N representation. The bootstrap interval includes zero. Descriptive verdict strings retained in the original code should be interpreted using the estimate and its interval; they do not establish equivalence or the absence of a shared error process.

| **Image setting** | **Adjusted odds ratio** | **95% CI** |
| --- | --- | --- |
| MIR images supplied | 0.3315 | 0.2071 to 0.5307 |
| EIR and FIR images withheld | 0.1131 | 0.0824 to 0.1552 |
| All four examinations pooled | 0.2050 | 0.1601 to 0.2627 |

# Source integrity

Each required file in the public deposit was verified byte for byte against the file used for the correction replay. The SHA-256 values below identify the original files. Line-ending conversion when copying source text from Word can change a file hash without changing its code; the repository files should therefore be copied directly from the archive.

analysis/common.py

bc9722f53c4242dda7d669520218ba782643d97081485566e33cfe50c38f3025

analysis/d1_robust.py

df654b0dba68c42887d9602fb70851df77dc5e46e9fdb81e554278ad5698c916

analysis/d1_psychometrics.py

12543b6d47e9b138cc8e6c9fab99442617b5881c537c9570bc2e63bbf071f092

analysis/out/long.csv

31693b62e63640da6eeda402fa5d6bd34dfccb74ac1c46b72fa624ca5693ebcc

analysis/out/item_master.csv

8b767ec3e18ea0b67ed608ab782d3f3e1a03da2d1387391387ebdd36158c1341

analysis/out/results_core.json

04b9bb1d07ccb2027c67b3cbe47599438ec9a1dd99c76e2c6270376b2690942f

analysis/out/results_d1.json

754d6e4bca324f7870fd536a651489f64b5806090828e37e11b978acb526a594

## Original correction script SHA-256

0468b13eb345a30e9b97ae6950438cae50ece14b9bc67745df52a62643731414

# Complete correction script

File path: revision2/qa/correct_declared_panel.py. The listing below preserves the full original source text and indentation. Its source comments and output field names are retained for provenance.

```
"""Reproduce archived estimators, restricting contaminated summaries to FRONT6 x 2.

No new model queries or estimators. Original analysis files remain read-only.
The original bootstrap RNG stream is retained, including preceding draws.
"""
from pathlib import Path
import hashlib
import json
import sys
import numpy as np
import pandas as pd
from scipy import stats
import statsmodels.formula.api as smf

ROOT = Path(__file__).resolve().parents[2]
sys.path.insert(0, str(ROOT / 'analysis'))
OUT = ROOT / 'analysis/out'
FRONT6 = ['Claude Opus 4.5', 'Claude Sonnet 4.5', 'Claude Haiku 4.5',
          'GPT-5.2 low', 'GPT-5.2 medium', 'GPT-5.2 high']
items = pd.read_csv(OUT / 'item_master.csv')
long = pd.read_csv(OUT / 'long.csv')
panel = long[long.model.isin(FRONT6) & long.prompt.isin(['neutral', 'expert'])].copy()
assert panel[['model', 'prompt']].drop_duplicates().shape[0] == 12
assert not panel.model.str.contains('Healthcademia').any()

# Execute only the original read-only prefix, ending before later analyses/writes.
robust_path = ROOT / 'analysis/d1_robust.py'
prefix = robust_path.read_text(encoding='utf-8').split('# ---------------- 4. trap gap bootstrap')[0]
original = {}
exec(compile(prefix, str(robust_path), 'exec'), original)
archive = json.loads((OUT / 'results_d1.json').read_text(encoding='utf-8'))
assert original['RB']['pillar2_plausibility_null'] == archive['robust2']['pillar2_plausibility_null']
read_line = 'long = pd.read_csv(os.path.join(OUT, "long.csv"))'
assert prefix.count(read_line) == 1
filtered_prefix = prefix.replace(read_line, read_line + '\nlong = long[long.model.isin(' + repr(FRONT6) + ') & long.prompt.isin(["neutral", "expert"])].copy()')
corrected = {}
exec(compile(filtered_prefix, str(robust_path), 'exec'), corrected)

# Reuse original error-concordance and entropy/confidence estimator block.
psych_path = ROOT / 'analysis/d1_psychometrics.py'
psych_source = psych_path.read_text(encoding='utf-8')
block = psych_source.split('# On trap items,')[1].split('# ---------------------------------------------------------------- save item master')[0]
block = '# On trap items,' + block
def concordance(frame):
    scope = {'mc': frame.copy(), 'items': items, 'offmap': dict(zip(items.uid, items.official)),
             'R': {}, 'np': np, 'pd': pd, 'stats': stats}
    exec(compile(block, str(psych_path), 'exec'), scope)
    return scope['R']
all_concordance = concordance(long)
for name in ['error_concordance', 'uncertainty_alignment']:
    assert all_concordance[name] == archive[name], name
panel_concordance = concordance(panel)
core = json.loads((OUT / 'results_core.json').read_text(encoding='utf-8'))
panel_displays = set(panel.display)
panel_cal = [v for v in core['calibration'] if v['display'] in panel_displays]
stable_cal = [v for v in panel_cal if v['stable']]
image_data = panel.merge(items[['uid', 'h_facility']], on='uid').dropna(subset=['h_facility'])
image_data['image'] = image_data.con_imagen.eq(1).astype(int)
image_checks = {}
for label, exams in [('MIR_supplied', ['MIR']), ('EIR_FIR_withheld', ['EIR', 'FIR']),
                     ('pooled_all_four', ['MIR', 'EIR', 'PIR', 'FIR'])]:
    frame = image_data[image_data.exam.isin(exams)]
    fit = smf.logit('correct ~ h_facility + image', frame).fit(disp=0)
    image_checks[label] = {'n': len(frame), 'exams': exams,
        'correct_and_total_by_image': frame.groupby('image').correct.agg(['sum', 'count']).to_dict('index'),
        'odds_ratio': float(np.exp(fit.params['image'])),
        'ci95': np.exp(fit.conf_int().loc['image']).tolist()}
assert round(image_checks['MIR_supplied']['odds_ratio'], 2) == .33
assert round(image_checks['EIR_FIR_withheld']['odds_ratio'], 2) == .11
assert round(image_checks['pooled_all_four']['odds_ratio'], 2) == .21
result = {
    'provenance': {'models': FRONT6, 'frames': ['neutral', 'expert'],
        'n_configurations': 12, 'n_responses': len(panel), 'seed': 20260621,
        'bootstrap_resampling_unit': 'individual error events, not items',
        'bootstrap_replicates': 2000, 'archived_unfiltered_estimators_reproduced': True,
        'source_sha256': {str(p.relative_to(ROOT)): hashlib.sha256(p.read_bytes()).hexdigest()
                          for p in [robust_path, psych_path, OUT/'long.csv', OUT/'item_master.csv']}},
    'old_plausibility': original['RB']['pillar2_plausibility_null'],
    'plausibility': corrected['RB']['pillar2_plausibility_null'],
    **panel_concordance,
    'error_events_by_model': panel[panel.correct.eq(0)].merge(items[['uid','h_popular']], on='uid').dropna(subset=['h_popular']).groupby('model').size().to_dict(),
    'calibration': {'n_frontier': len(panel_cal), 'n_stable_frontier': len(stable_cal),
                    'underconfident': [v['display'] for v in stable_cal if v['overconfidence'] < 0],
                    'ece_min_max': [min(v['ece'] for v in panel_cal), max(v['ece'] for v in panel_cal)],
                    'archived_stable_nonfrontier': [v['display'] for v in core['calibration'] if v['stable'] and v['display'] not in panel_displays]},
    'latency_frontier': [v for v in core['latency'] if v['display'] in panel_displays]
    ,'image_scope_verification': image_checks
}
target = Path(__file__).with_name('corrected_numbers.json')
target.write_text(json.dumps(result, ensure_ascii=False, indent=2), encoding='utf-8')
print(json.dumps({k: result[k] for k in ['plausibility', 'error_concordance', 'uncertainty_alignment', 'calibration']}, indent=2))
print(target)
```
